# Beverage consumption and mortality among adults with type 2 diabetes: prospective cohort study

**DOI:** 10.1136/bmj-2022-073406

**Published:** 2023-04-19

**Authors:** Le Ma, Yang Hu, Derrick J Alperet, Gang Liu, Vasanti Malik, JoAnn E Manson, Eric B Rimm, Frank B Hu, Qi Sun

**Affiliations:** 1 Department of Nutrition, Harvard TH Chan School of Public Health, Boston, MA 02115, USA; 2 School of Public Health, Xi’an Jiaotong University Health Science Center, Xi’an, China; 3 Key Laboratory of Environment and Genes Related to Diseases (Xi’an Jiaotong University), Ministry of Education of China, Xi’an, China; 4 Department of Nutrition and Food Hygiene, Hubei Key Laboratory of Food Nutrition and Safety, Ministry of Education Key Laboratory of Environment and Health, School of Public Health, Tongji Medical College, Huazhong University of Science and Technology, Wuhan, China; 5 Department of Nutritional Sciences, Temerty Faculty of Medicine, University of Toronto, Toronto, Ontario, Canada; 6 Department of Epidemiology, Harvard T.H. Chan School of Public Health, Boston, Massachusetts, USA; 7 Channing Division of Network Medicine, Department of Medicine, Brigham and Women’s Hospital and Harvard Medical School, Boston, Massachusetts, USA; 8 Department of Medicine, Brigham and Women’s Hospital, Harvard Medical School, Boston, Massachusetts, USA; 9 Division of Preventive Medicine, Department of Medicine, Brigham and Women’s Hospital and Harvard Medical School, Boston, Massachusetts, USA

## Abstract

**Objective:**

To investigate the intake of specific types of beverages in relation to mortality and cardiovascular disease (CVD) outcomes among adults with type 2 diabetes.

**Design:**

Prospective cohort study.

**Setting:**

Health professionals in the United States.

**Participants:**

15 486 men and women with a diagnosis of type 2 diabetes at baseline and during follow-up (Nurses’ Health Study: 1980-2018; and Health Professionals Follow-Up Study: 1986-2018). Beverage consumption was assessed using a validated food frequency questionnaire and updated every two to four years.

**Main outcome measures:**

The main outcome was all cause mortality. Secondary outcomes were CVD incidence and mortality.

**Results:**

During an average of 18.5 years of follow-up, 3447 (22.3%) participants with incident CVD and 7638 (49.3%) deaths were documented. After multivariable adjustment, when comparing the categories of lowest intake of beverages with the highest intake, the pooled hazard ratios for all cause mortality were 1.20 (95% confidence interval 1.04 to 1.37) for sugar sweetened beverages (SSBs), 0.96 (0.86 to 1.07) for artificially sweetened beverages (ASBs), 0.98 (0.90 to 1.06) for fruit juice, 0.74 (0.63 to 0.86) for coffee, 0.79 (0.71 to 0.89) for tea, 0.77 (0.70 to 0.85) for plain water, 0.88 (0.80 to 0.96) for low fat milk, and 1.20 (0.99 to 1.44) for full fat milk. Similar associations were observed between the individual beverages and CVD incidence and mortality. In particular, SSB intake was associated with a higher risk of incident CVD (hazard ratio 1.25, 95% confidence interval 1.03 to 1.51) and CVD mortality (1.29, 1.02 to 1.63), whereas significant inverse associations were observed between intake of coffee and low fat milk and CVD incidence. Additionally, compared with those who did not change their consumption of coffee in the period after a diabetes diagnosis, a lower all cause mortality was observed in those who increased their consumption of coffee. A similar pattern of association with all cause mortality was also observed for tea, and low fat milk. Replacing SSBs with ABSs was significantly associated with lower all cause mortality and CVD mortality, and replacing SSBs, ASBs, fruit juice, or full fat milk with coffee, tea, or plain water was consistently associated with lower all cause mortality.

**Conclusions:**

Individual beverages showed divergent associations with all cause mortality and CVD outcomes among adults with type 2 diabetes. Higher intake of SSBs was associated with higher all cause mortality and CVD incidence and mortality, whereas intakes of coffee, tea, plain water, and low fat milk were inversely associated with all cause mortality. These findings emphasize the potential role of healthy choices of beverages in managing the risk of CVD and premature death overall in adults with type 2 diabetes.

**Figure:**
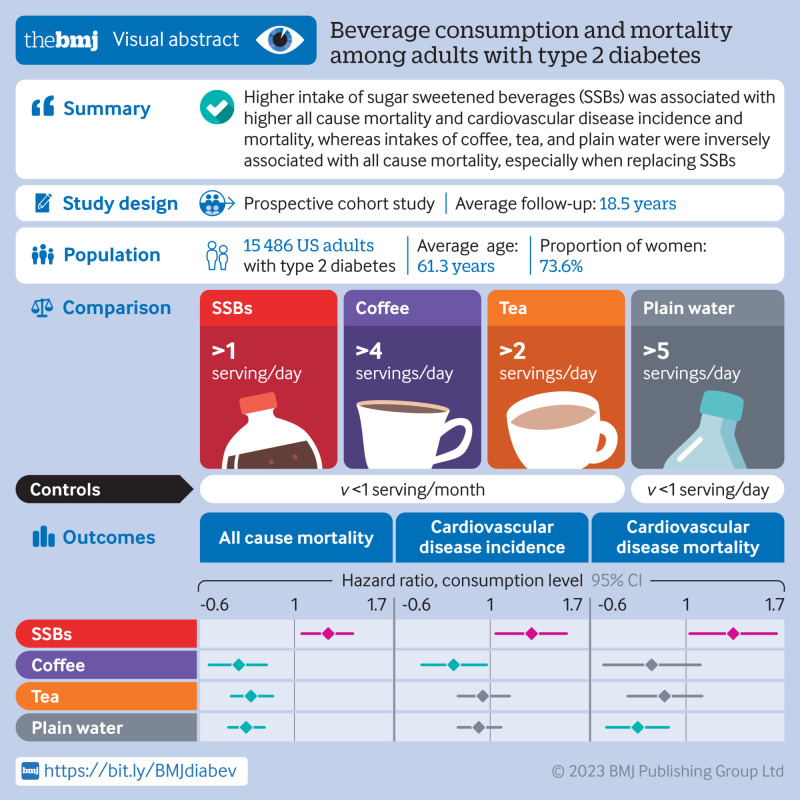


## Introduction

In 2021 about 537 million adults worldwide had diabetes and this number is projected to rise to 783 million by 2045.^1^ The risk of cardiovascular disease (CVD), other morbidities, and premature death is particularly increased in adults with type 2 diabetes.^2^ Dietary interventions play a fundamental role in the glycemic management of adults with type 2 diabetes, although the prevailing dietary recommendations and nutritional guidelines for the general population may not necessarily be directly relevant to adults with diabetes because of their altered metabolism of carbohydrates and other macronutrients.^3^ ^4^ It is therefore important to evaluate various dietary intakes, such as beverages, in relation to disease outcomes and mortality among adults with diabetes.

That different types of beverages may have distinct health effects depending on the contents of sugar and other constituents has been well documented.^5^ ^6^ Several meta-analyses of prospective cohort studies have shown that high intake of beverages with a low energy density, such as plain water, low fat milk, and coffee was clearly associated with a lower incidence of obesity, type 2 diabetes, CVD, and all cause and cause specific mortality, primarily in the general population.^7^ ^8^ ^9^ In contrast with these findings, intake of sugar sweetened beverages (SSBs) was associated with a higher risk of type 2 diabetes and CVD.^10^ ^11^ As a potential alternative to SSBs, artificially sweetened beverages (ASBs) contain little to no sugar or calories. The American Heart Association and American Diabetes Association have recommended that non-nutritive sweeteners could replace added sugar within a balanced diet to maintain a healthy weight and minimize cardiometabolic risk.^12^ To date, the association of individual beverage consumption with risk of CVD and mortality among adults with type 2 diabetes remains largely unexplored. Nonetheless, several clinical trials have shown some favorable effects of intakes of ASBs, coffee, or tea on body weight, lipid profile, and cardiometabolic risk factors among adults with type 2 diabetes.^13^ ^14^ ^15^

To fill this knowledge gap, we prospectively investigated individual beverage consumption after a diagnosis of type 2 diabetes mellitus, as well as changes in individual beverage consumption before and after the diagnosis, in relation to subsequent risk of CVD and mortality among adults with type 2 diabetes participating in the Nurses’ Health Study and Health Professionals Follow-Up Study in the United States. We also estimated associations between substituting one beverage for another and CVD risk and mortality.

## Methods

### Study population

The Nurses’ Health Study, a prospective cohort study initiated in 1976, enrolled 121 700 female registered nurses aged 30 to 55 years.^16^ The Health Professionals Follow-Up Study cohort was established in 1986 and enrolled 51 529 male health professionals aged 40 to 75 years.^17^ In both studies, detailed information on dietary and lifestyle factors, medical history, and disease status was collected at baseline and updated every two to four years through validated questionnaires.^18^ The cumulative response rate exceeded 90% for both cohorts. In the current analysis, we included participants with prevalent type 2 diabetes at baseline (1980 for the Nurses’ Health Study, and 1986 for the Health Professionals Follow-Up Study, when dietary information was first collected using a validated food frequency questionnaire), as well as participants with a diagnosis of incident type 2 diabetes during follow-up to 2018. We excluded participants if they had type 1 diabetes, CVD, or cancer at baseline; reported CVD or cancer before the diagnosis of type 2 diabetes during follow-up; left more than nine blank responses on the food frequency questionnaire or reported implausible daily energy intakes (<2510 or >14 644 kJ/day for women, and <3347 or >17 573 kJ/day for men); or had incomplete information on beverage consumption or dietary data at diabetes diagnosis. After exclusions, a total of 11 399 participants of the Nurses’ Health Study and 4087 participants of the Health Professionals Follow-Up Study with type 2 diabetes were included in the current analysis. For the analysis of changes in beverage consumption from before to after the diabetes diagnosis, we further excluded participants with type 2 diabetes at baseline or those with missing data on beverage consumption assessed before the diabetes diagnosis (n=2715), which left 9252 women and 3519 men for the change analysis.

### Assessment of beverage intake

Intake of beverages was assessed using the validated food frequency questionnaires administered every two to four years. Participants were asked how often, on average (never to >6 times per day), they had consumed SSBs, ASBs, fruit juice, coffee, tea, low fat milk (skimmed, 1% or 2% fat), full fat milk, or plain water of a prespecified portion size (cup, glass, can, or bottle). SSBs included caffeinated colas, caffeine-free colas, other carbonated SSBs, and non-carbonated SSBs (fruit punches, lemonades, or other fruit drinks). ASBs included low calorie cola with caffeine, low calorie caffeine-free cola, and other low calorie beverages. Fruit juices included orange, apple, grapefruit, or other fruit juices. Coffee included caffeinated and decaffeinated varieties. A validation study conducted among a subsample of the participants in the Health Professionals Follow-Up Study showed reasonable validity for the assessment of beverage intake.^19^ The correlation coefficients between the food frequency questionnaire and multiple diet records were 0.84 for colas, 0.73 for low calorie colas, 0.75-0.89 for fruit juices, 0.93 for coffee, 0.77 for tea, 0.88 for low fat milk, 0.67 for full fat milk, and 0.52 for plain water.^19^ Similar correlation coefficients were found in a validation study conducted among a subsample of participants in the Nurses’ Health Study.^20^ Our primary dietary factors of interest were specific types of beverage consumption assessed after the diabetes diagnosis, and changes in beverage consumption before and after the diagnosis. We assessed the pre-diabetes beverage intake from the most proximal questionnaires before diabetes was ascertained.

### Ascertainment of type 2 diabetes

Participants who reported a physician diagnosis of diabetes mellitus in the biennial questionnaires were sent a validated supplementary questionnaire about diagnostic tests, symptoms, and hypoglycemic treatment. The National Diabetes Data Group and American Diabetes Association criteria were applied to ascertain a diagnosis of type 2 diabetes (see supplementary appendix).^21^ ^22^ We excluded from the current analysis those participants who reported a diagnosis of type 1 diabetes on the supplementary questionnaire. Studies among 62 participants in the Nurses’ Health Study and 59 participants in the Health Professionals Follow-Up Study showed high validity in the supplementary questionnaire, with 98% and 97% of questionnaire confirmed type 2 diabetes diagnoses validated by medical record review in these women and men, respectively.^23^ ^24^

### Ascertainment of outcomes

The primary endpoint was all cause mortality. We also examined the secondary outcomes of CVD incidence and mortality. Deaths were identified from reports by the next of kin or postal authorities or from searches of the National Death Index (see supplementary appendix).^25^ ICD-9 (international classification of diseases, ninth revision) codes were used to classify deaths from CVD (codes 390-459), cancer (codes 140-208.32), or other causes. Incident CVD was defined as fatal and non-fatal coronary heart disease, including coronary artery bypass graft surgery and non-fatal myocardial infarction, and as fatal and non-fatal stroke (see supplementary appendix).

### Assessment of covariates

In both cohorts, information on lifestyle factors and medical history was collected at baseline and in biennial questionnaires. The supplementary appendix provides details of the assessments of covariates. To assess overall diet quality, we calculated the Alternate Healthy Eating Index (AHEI) score based on intakes of 11 foods and nutrients predictive of chronic disease risk, including vegetables, fruits, whole grains, SSBs and fruit juice, nuts and legumes, red and processed meat, trans fatty acids, long chain omega 3 fatty acids, other polyunsaturated fats, sodium, and alcohol.^26^ In the current study, we modified the AHEI score by excluding the consumption of SSBs and fruit juices.

### Statistical analysis

The Kolmogorov-Smirnov normality test was used to assess distributions of continuous variables for normality, and natural logarithm transformations of skewed variables were applied before analyses. In descriptive analyses, continuous variables were expressed as means (standard deviations) for normally distributed variables or medians (interquartile ranges) for skewed variables, and categorical variables were represented by frequency and percentage. General linear models were used to calculate mean characteristics of the study participants at the time of diabetes diagnosis, and a test for linear trend using the Wald test was performed by assigning the median value to each category of beverage consumption and modeling this variable as a continuous variable.

For each participant, we calculated person years of follow-up from the date of diabetes diagnosis to the date of occurrence of study outcomes, last return of a valid follow-up questionnaire, or end of follow-up (30 June 2018 for the Nurses’ Health Study, and 30 January 2018 for the Health Professionals Follow-Up Study), whichever came first. Because changes in diet after a diagnosis of cancer could distort the associations of interest, for the CVD incidence analyses we stopped updating dietary variables after participants reported a diagnosis of cancer. For mortality analyses, dietary intake was not updated after a diagnosis of cancer or CVD. Time varying Cox proportional hazards models, conditioned on age and follow-up cycle, were applied to estimate hazard ratios and 95% confidence intervals for the associations of each beverage intake with all cause mortality, CVD incidence, and CVD mortality. Changes in beverage intake from before to after the diabetes diagnosis were defined as the absolute difference in beverage consumption (time varying post-diabetes beverage intake minus pre-diabetes beverage intake). The time varying covariates assessed during follow-up were considered in the multivariable models. Missing data for beverage consumptions and covariates during follow-up were replaced by the most recent valid assessments. In the multivariable model, we adjusted for age (years), duration of diabetes (years), sex (men or women), white ethnicity (yes or no), physical activity (<3.0, 3.0-8.9, 9.0-17.9, 18.0-26.9, ≥27.0 metabolic equivalents of task-hours/week), smoking status (never, former, current 1-14 cigarettes/day, current ≥15 cigarettes/day), alcohol consumption (0, 0.1-4.9, 5.0-14.9, ≥15.0 g/day), menopausal status and post-menopausal hormone use (pre-menopause, post-menopause (never, former, or current hormone use), or missing; Nurses’ Health Study only), family history of type 2 diabetes (yes or no) or myocardial infarction (yes or no), intake of total energy, and modified AHEI score (all in fourths). To further reduce the impact of confounding by existing comorbidities, disease management, and weight change, we further included history of hypertension (yes or no) or hypercholesterolemia (yes or no), use of antihypertensive (yes or no) or lipid lowering drug (yes or no), aspirin use (yes or no), diabetes drug use (oral drug only, insulin use, or others), and change in body mass index (BMI) before to after the diabetes diagnosis in the fully adjusted model.^27^ We mutually adjusted for different types of beverage intakes in the analysis of specific types of beverages. To obtain overall estimates for men and women and to increase statistical power, we pooled the hazard ratios from each model from the two cohorts with the use of an inverse variance weighted meta-analysis by the random effects model, which accounted for between study heterogeneity.^28^ CVD incidence and mortality were also examined according to the per serving intake of beverages. In the analysis of changes in beverage consumption from before to after the diabetes diagnosis, we further adjusted for beverage intake before the diagnosis in the multivariable model.

In the current study, we tested the proportional hazards assumption by using a likelihood ratio test comparing models with and without multiplicative interaction terms between beverage consumptions and calendar year, and we did not find evidence of violation of the assumption. Tests for trend were performed by assigning a median value to each beverage consumption category as a continuous variable. To examine the dose-response relationships between beverage intake and the outcomes, we used restricted cubic spline regression with three knots. Tests for non-linearity were based on the likelihood ratio test comparing two models: one with only the linear term and the other with the linear and the cubic spline terms.

We estimated the association of substituting a serving of one beverage for another by including both as continuous variables in the same multivariable model. Differences in their β coefficients were used to calculate the hazard ratios for the substitution effects, and their variances and covariance matrix were used to derive the 95% confidence intervals for the point estimate.

Several sensitivity analyses were conducted to test the robustness of our findings. First, we restricted our analyses to adults with incident type 2 diabetes by excluding those with prevalent diabetes at baseline. Second, we excluded deaths that occurred within four years after the diabetes diagnosis to examine whether the results were impacted by reverse causation bias. Third, a four year and eight year lag were placed between the assessment of beverage intake and outcome incidence, respectively. In these analyses, beverage intake was used to predict disease occurring four years or eight years later. Fourth, given that weight change can be an intermediate outcome, in our final model we adjusted for BMI before the diabetes diagnosis, instead of change in BMI before to after the diagnosis to examine the robustness of our observed associations. Fifth, we examined potential confounding from measures of socioeconomic status by adding partner’s education and self-rated socioeconomic status to the final model. Sixth, we used beverage intake assessed before the diabetes diagnosis instead of the cumulative average after diagnosis to evaluate whether changes in consumption pattern immediately after the diagnosis might impact the associations of interest. Seventh, as it is likely that participants might quit drinking unhealthy drinks immediately after the diabetes diagnosis, we skipped the first food frequency questionnaire after the diagnosis and used the rest to calculate cumulative averages and re-examine the associations. Eighth, we also conducted a sensitivity analysis excluding current and former smokers to further reduce confounding by smoking status. Ninth, we performed an analysis restricted to adults with asymptomatic type 2 diabetes to assess the impact of diabetes screening on associations of interest. Tenth, we controlled for the number of diabetes related symptoms as a measure of disease severity. Lastly, to reduce potential confounding by glucose control, we further adjusted for the self-reported levels of glycated hemoglobin HbA_1c_ in a subset of the participants (n=5192).

All statistical analyses were performed with SAS software, version 9.4 (SAS Institute, Cary, NC). Two sided P<0.05 was considered statistically significant.

### Patient and public involvement

Participants were not involved in the design, development of outcome measures, or other aspects of the conduct of the study. Participants are given updates on main findings of the cohort studies through newsletters and the study websites (https://www.nurseshealthstudy.org and https://www.hsph.harvard.edu/hpfs/).

## Results

Table 1 shows the baseline characteristics of the study participants at diagnosis of type 2 diabetes and according to consumption of different beverages. Participants with higher intakes of SSBs were younger and more likely to have a higher consumption of total energy than participants with lower intakes. Those who consumed more ASBs also had a higher BMI at diabetes diagnosis (P for trend <0.001). Greater coffee consumption was positively associated with smoking. Participants who consumed a higher volume of low fat milk and plain water were more likely to use aspirin, antihypertensives, and lipid lowering drugs. Individual beverages were weakly correlated with each other; the highest Spearman correlation coefficients were 0.25 between low fat milk and plain water and 0.19 between low fat milk and fruit juice (see supplementary figure 1).

**Table 1:** Baseline characteristics of adults with type 2 diabetes according to consumption of different beverages.* Values are numbers (percentages) unless stated otherwise

Characteristics	SSBs			ASBs			Fruit juice			Coffee			Plain water			Low fat milk	
	<1 serving/month	>1 serving/day		<1 serving/month	>2 servings/day		<1 serving/month	>1 serving/day		<1 serving/month	>4 servings/day		<1 serving/day	>5 servings/day		<1 serving/day	>1 serving/day
No of participants	8142	1152		6918	1527		4545	2103		6618	1086		4891	2182		5225	2131
Mean (SD) age† (years)	61.6 (9.8)	58.2 (10.2)		62.5 (9.9)	56.7 (9.4)		61.9 (9.3)	62.9 (9.4)		61.6 (9.8)	57.1 (9.9)		60.7 (10.1)	61.7 (9.0)		60.7 (10.1)	61.7 (9.0)
Men	2005 (24.6)	298 (25.9)		1985 (28.7)	326 (21.4)		1202 (26.5)	734 (34.9)		1964 (26.7)	255 (23.5)		1511 (309)	385 (17.6)		1544 (29.6)	522 (24.5)
White ethnicity	7752 (95.2)	1077 (93.5)		6492 (93.8)	1480 (96.9)		4279 (94.2)	2011 (95.6)		6222 (94.0)	1068 (98.3)		4567 (93.4)	2077 (95.2)		4842 (92.7)	2072 (97.2)
Mean (SD) BMI	30.2 (6.0)	30.8 (6.0)		29.7 (5.9)	32.2 (6.1)		30.6 (6.0)	29.9 (5.6)		30.4 (6.1)	29.4 (5.7)		30.2 (5.8)	31.1 (6.1)		30.2 (5.8)	31.1 (6.1)
Mean (SD) MET-hours/week	17.4 (26.1)	16.3 (30.2)		17.2 (27.6)	16 (25.7)		16.8 (25.2)	19.4 (29.8)		17.5 (28.2)	18.5 (29.8)		17.4 (29.1)	18.0 (24.6)		17.4 (29.1)	18.0 (24.6)
Current smoker	1084 (13.3)	180 (15.6)		983 (14.2)	234 (15.3)		603 (13.3)	200 (9.5)		674 (10.2)	383 (35.3)		728 (14.9)	215 (9.9)		867 (16.6)	238 (11.2)
Former smoker	3536 (43.4)	425 (36.9)		2734 (39.5)	659 (43.2)		1923 (42.3)	865 (41.1)		2537 (38.3)	376 (34.6)		1914 (39.1)	944 (43.3)		1992 (38.1)	832 (39.0)
Postmenopausal‡	5094 (83.0)	626 (73.7)		4176 (84.7)	857 (71.4)		2882 (86.2)	1156 (84.4)		4022 (82.9)	582 (70.0)		2687 (79.5)	1533 (85.3)		2898 (78.7)	1315 (81.7)
Ever menopausal hormone use‡	2803 (45.7)	355 (41.6)		2213 (44.9)	490 (40.8)		1519 (45.4)	657 (48.0)		2235 (46.0)	276 (33.2)		1287 (38.1)	938 (52.2)		1325 (36.0)	797 (49.5)
Hypertension	5123 (62.9)	683 (59.2)		4263 (61.6)	945 (61.9)		2857 (62.9)	1389 (66.1)		4226 (63.9)	494 (45.5)		2919 (59.7)	1469 (67.3)		3077 (58.9)	1365 (64.1)
Hypercholesterolemia	4167 (51.2)	547 (47.5)		3525 (51.0)	715 (46.8)		2452 (54.0)	1088 (51.7)		3452 (52.2)	381 (35.1)		2378 (48.6)	1182 (54.2)		2349 (45.0)	1072 (50.3)
Family history of MI	1791 (22.0)	630 (21.2)		1455 (21.0)	346 (22.7)		1003 (22.1)	499 (23.7)		1422 (21.5)	233 (21.5)		1081 (22.1)	450 (20.6)		1131 (21.7)	444 (20.8)
Use of antihypertensive	2730 (33.5)	395 (34.3)		2270 (32.8)	500 (32.7)		1442 (31.7)	897 (42.7)		2238 (33.8)	220 (20.3)		1286 (26.3)	950 (43.5)		1296 (24.8)	891 (41.8)
Use of lipid lowering drug	1473 (18.1)	195 (16.9)		1158 (16.7)	223 (14.6)		801 (17.6)	401 (19.1)		1111 (16.8)	124 (11.4)		670 (13.7)	454 (20.8)		611 (11.7)	395 (18.5)
Aspirin use	4064 (49.9)	618 (53.7)		3364 (48.6)	779 (51.0)		2079 (45.7)	1190 (56.6)		3152 (47.6)	576 (53.0)		2311 (47.3)	1195 (54.8)		2431 (46.5)	1183 (55.5)
Mean (SD) total energy intake (kcal/day)	1685.6 (557.9)	2246.7 (640.9)		1815.7 (607.9)	1853.1 (603.7)		1711.3 (591.4)	2049.2 (596.8)		1764.8 (590.8)	1901.9 (627.9)		1760.9 (603.3)	1844.3 (610.9)		1760.9 (603.3)	1844.3 (610.9)
Alcohol consumption (g/day)	0.9 (0-5.8)	0.8 (0-3.4)		0.9 (0-5.8)	0.8 (0-3.4)		0.9 (0-4.9)	1.2 (0-6.0)		0.0 (0-3.1)	1.0 (0-5.6)		1.0 (0-5.6)	0.0 (0-3.5)		1.0 (0-6.0)	0.0 (0-3.5)
Mean (SD) modified AHEI score	49.9 (10.8)	44.2 (9.7)		48.0 (10.7)	47.3 (10.3)		48.8 (11.0)	49.5 (10.3)		48.7 (10.6)	46.8 (10.7)		47.7 (11.0)	47.9 (10.0)		47.7 (11.0)	47.9 (10.0)

AHEI=Alternative Healthy Eating Index; ASBs=artificially sweetened beverages; BMI=body mass index; MET=metabolic equivalent of task; MI=myocardial infarction; SSBs=sugar sweetened beverages.

* Values standardized to age distribution of the study population.

† Values were not age adjusted.

‡ Women only.

**Fig 1:**
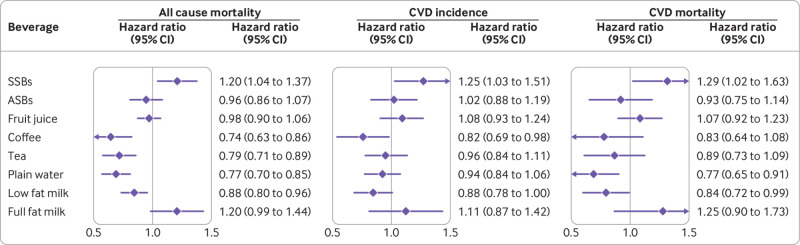
Hazard ratios (95% CIs) of all cause mortality, CVD incidence, and CVD mortality according to consumption of specific types of beverages among adults with type 2 diabetes. Hazard ratios for CVD incidence comparing extreme categories of consumption of specific types of beverages (SSB: <1 serving/month *v* >1 serving/day; ASB: <1 serving/month *v* >2 servings/day; fruit juice: <1 serving/month *v* >1 serving/day; coffee: <1 serving/month *v* >4 servings/day; tea: <1 serving/month *v* >2 servings/day; plain water: <1 serving/day *v* >5 servings/day; low fat milk: <1 serving/month *v* >2 servings/day; full fat milk: <1 serving/month *v* >1 serving/day) among individuals with type 2 diabetes were adjusted for age (continuous), duration of diabetes mellitus (years), sex (men or women), white ethnicity (yes or no), physical activity (<3.0, 3.0-8.9, 9.0-17.9, 18.0-26.9, ≥27.0 metabolic equivalents of task-hours/week), smoking status (never, former, current 1-14 cigarettes/day, current ≥15 cigarettes/day), alcohol consumption (0, 0.1-4.9, 5.0-14.9, ≥15.0 g/day), menopausal status and post-menopausal hormone use (pre-menopause, post-menopause (never, former, or current hormone use), or missing; Nurses’ Health Study only), family history of type 2 diabetes (yes or no) or myocardial infarction (yes or no), intake of total energy (continuous), the modified Alternative Healthy Eating Index score (fourths), history of hypertension (yes or no) or hypercholesterolemia (yes or no), use of antihypertensive (yes or no) or lipid lowering drug (yes or no), aspirin use (yes or no), diabetes drug use (oral drug only, insulin use, or others), and change in body mass index before to after diabetes diagnosis. Individual beverage consumption was mutually adjusted. ASB=artificially sweetened beverage; CI=confidence interval; CVD=cardiovascular disease; SSB=sugar-sweetened beverage

**Fig 2:**
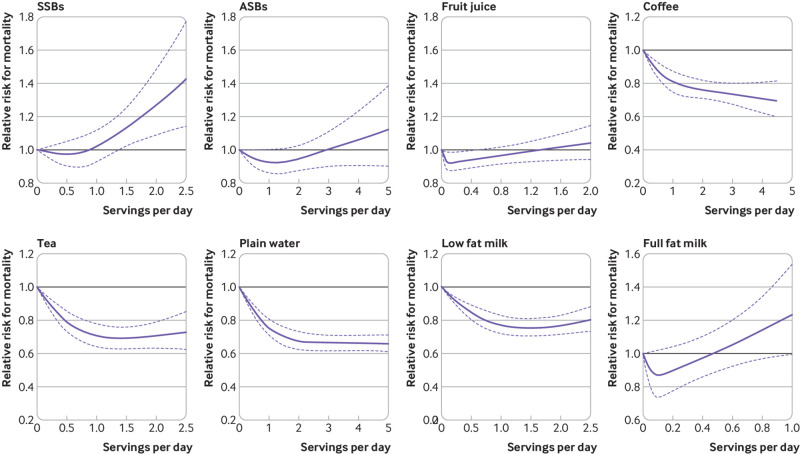
Restricted cubic spline analysis of association between individual beverage consumption and all cause mortality among adults with type 2 diabetes. Adjusted for age (continuous), duration of diabetes (years), sex (men or women), white ethnicity (yes or no), physical activity (<3.0, 3.0-8.9, 9.0-17.9, 18.0-26.9, ≥27.0 metabolic equivalents of task-hours/week), smoking status (never, former, current 1-14 cigarettes/day, current ≥15 cigarettes/day), alcohol consumption (0, 0.1-4.9, 5.0-14.9, ≥15.0 g/day), menopausal status and post-menopausal hormone use (pre-menopause, post-menopause (never, former, or current hormone use), or missing; Nurses’ Health Study only), family history of type 2 diabetes (yes or no) or myocardial infarction (yes or no), intake of total energy (continuous), the modified Alternative Healthy Eating Index score (fourths), history of hypertension (yes or no) or hypercholesterolemia (yes or no), use of antihypertensive (yes or no) or lipid lowering drug (yes or no), aspirin use (yes or no), diabetes drug use (oral drug only, insulin use, or others), and change in body mass index before to after diabetes diagnosis. Individual beverage consumption was mutually adjusted. ASB=artificially sweetened beverage; CI=confidence interval; CVD=cardiovascular disease; HR=hazard ratio; SSB=sugar sweetened beverage

We documented 7638 (49.3%) deaths during 285 967 person years of follow-up. The pooled hazard ratio for all cause mortality in participants with the lowest intake of SSB compared with highest intake of SSB was 1.20 (95% confidence interval 1.04 to 1.37) (fig 1 and table 2). In contrast, high intakes of certain beverages were associated with lower mortality: 0.74 (0.63 to 0.86) for coffee, 0.79 (0.71 to 0.89) for tea, 0.77 (0.70 to 0.85) for plain water, and 0.88 (0.80 to 0.96) for low fat milk. We did not observe clear association patterns for other beverages, such as ASBs, fruit juice, or full fat milk. The associations of intake of specific types of beverages with CVD incidence and mortality were also evaluated separately, and the pattern of associations was largely similar to those for all cause mortality (fig 1 and supplementary tables 1 and 2). Results from the multivariable adjusted restricted cubic spline regression showed that the risk of all cause mortality decreased non-linearly with increasing intake of coffee, tea, plain water, and low fat milk (all P for non-linearity <0.001), whereas the association between SSBs and all cause mortality was more linear (fig 2). Each one serving/day increment in SSBs was associated with 8% (95% confidence interval 2% to 14%) higher all cause mortality.

**Table 2:** Hazard ratios for all cause mortality according to consumption of specific types of beverages among adults with type 2 diabetes

	Hazard ratio (95% CI) by consumption level					Every 1 serving/day	P value for trend
**SSBs**	**<1 serving/month**	**<1 serving/week**	**1-3 servings/week**	**4-7 servings/week**	**>1 serving/day**		
No of participants/person years	5669/200 809	942/40 299	485/21 511	304/13 109	238/10 239		
Age adjusted model	1	0.84 (0.79 to 0.90)	0.85 (0.77 to 0.93)	0.93 (0.83 to 1.05)	1.11 (0.97 to 1.26)	1.03 (0.98 to 1.09)	0.65
Multivariable model*	1	0.96 (0.89 to 1.03)	0.99 (0.90 to 1.09)	1.01 (0.90 to 1.14)	1.19 (1.04 to 1.36)	1.07 (1.02 to 1.13)	0.02
Fully adjusted model†	1	0.97 (0.91 to 1.05)	1.01 (0.92 to 1.11)	1.02 (0.91 to 1.15)	1.20 (1.04 to 1.37)	1.08 (1.02 to 1.14)	0.01
**ASBs**	**<1 serving/month**	**<3 servings/week**	**3-6 servings/week**	**1-2 servings/day**	**>2 servings/day**		
No of participants/person years	3868/117 911	1216/49 777	1269/54 548	698/34 472	587/29 260		
Age adjusted model	1	0.74 (0.70 to 0.79)	0.83 (0.77 to 0.88)	0.89 (0.82 to 0.96)	1.16 (1.06 to 1.26)	1.05 (1.02 to 1.08)	0.002
Multivariable model*	1	0.78 (0.72 to 0.83)	0.82 (0.76 to 0.89)	0.80 (0.73 to 0.89)	0.91 (0.81 to 1.01)	0.98 (0.94 to 1.01)	0.04
Fully adjusted model†	1	0.83 (0.77 to 0.89)	0.88 (0.81 to 0.94)	0.85 (0.77 to 0.94)	0.96 (0.86 to 1.07)	0.98 (0.94 to 1.01)	0.34
**Fruit juice**	**<1 serving/month**	**<1 serving/week**	**1-3 servings/week**	**4-7 servings/week**	**>1 serving/day**		
No of participants/person years	3284/103 402	944/42 993	928/40 754	1321/54 625	1161/44 193		
Age adjusted model	1	0.80 (0.75 to 0.86)	0.86 (0.80 to 0.93)	0.83 (0.78 to 0.89)	0.93 (0.86 to 1.01)	0.99 (0.96 to 1.03)	<0.001
Multivariable model*	1	0.84 (0.78 to 0.90)	0.90 (0.83 to 0.97)	0.86 (0.80 to 0.92)	0.96 (0.88 to 1.04)	1.00 (0.96 to 1.04)	0.03
Fully adjusted model†	1	0.87 (0.81 to 0.93)	0.92 (0.86 to 1.00)	0.89 (0.83 to 0.95)	0.98 (0.90 to 1.06)	1.01 (0.97 to 1.05)	0.12
**Coffee**	**<1 serving/month**	**<1 serving/day**	**1-2 servings/day**	**3-4 servings/day**	**>4 servings/day**		
No of participants/person years	4897/138 144	833/39 261	872/41 830	852/53 379	184/13 354		
Age adjusted model	1	0.61 (0.57 to 0.66)	0.57 (0.53 to 0.62)	0.51 (0.48 to 0.55)	0.60 (0.52 to 0.70)	0.81 (0.79 to 0.83)	<0.001
Multivariable model*	1	0.81 (0.75 to 0.87)	0.79 (0.73 to 0.85)	0.69 (0.64 to 0.74)	0.71 (0.61 to 0.82)	0.90 (0.87 to 0.92)	<0.001
Fully adjusted model†	1	0.84 (0.78 to 0.91)	0.83 (0.77 to 0.90)	0.72 (0.67 to 0.78)	0.74 (0.63 to 0.86)	0.91 (0.89 to 0.93)	<0.001
**Tea**	**<1 serving/month**	**<3 servings/week**	**3-6 servings/week**	**1-2 servings/day**	**>2 servings/day**		
No of participants/person years	5473/158 479	871/51 426	615/33 659	365/21 457	314/20 947		
Age adjusted model	1	0.84 (0.78 to 0.91)	0.83 (0.77 to 0.90)	0.72 (0.67 to 0.78)	0.74 (0.63 to 0.86)	0.90 (0.88 to 0.93)	<0.001
Multivariable model*	1	0.57 (0.53 to 0.61)	0.58 (0.53 to 0.63)	0.61 (0.55 to 0.68)	0.60 (0.53 to 0.67)	0.78 (0.74 to 0.82)	<0.001
Fully adjusted model†	1	0.79 (0.74 to 0.86)	0.84 (0.77 to 0.92)	0.83 (0.74 to 0.92)	0.79 (0.71 to 0.89)	0.94 (0.90 to 0.97)	<0.001
**Plain water**	**<1 serving/day**	**1 serving/day**	**2-3 servings/day**	**3-5 servings/day**	**>5 serving/day**		
No of participants/person years	4246/103 523	486/24 751	1374/67 553	960/54 188	572/35 953		
Age adjusted model	1	0.58 (0.53 to 0.64)	0.51 (0.48 to 0.54)	0.45 (0.42 to 0.48)	0.48 (0.44 to 0.52)	0.84 (0.83 to 0.85)	<0.001
Multivariable model*	1	0.79 (0.72 to 0.87)	0.75 (0.70 to 0.80)	0.69 (0.64 to 0.75)	0.74 (0.68 to 0.82)	0.93 (0.92 to 0.95)	<0.001
Fully adjusted model†	1	0.84 (0.76 to 0.92)	0.79 (0.74 to 0.85)	0.73 (0.68 to 0.79)	0.77 (0.70 to 0.85)	0.94 (0.93 to 0.96)	<0.001
**Low fat milk**	**<1 serving/month**	**<3 servings/week**	**3-6 servings/week**	**1-2 servings/day**	**>2 servings/day**		
No of participants/person years	4138/103 028	616/32 359	1177/61 638	1050/53 761	657/35 183		
Age adjusted model	1	0.53 (0.48 to 0.57)	0.50 (0.47 to 0.53)	0.51 (0.48 to 0.55)	0.52 (0.47 to 0.56)	0.79 (0.76 to 0.81)	<0.001
Multivariable model*	1	0.81 (0.74 to 0.89)	0.80 (0.74 to 0.86)	0.83 (0.77 to 0.89)	0.83 (0.75 to 0.91)	0.96 (0.93 to 0.99)	<0.001
Fully adjusted model†	1	0.86 (0.79 to 0.94)	0.85 (0.79 to 0.92)	0.87 (0.81 to 0.94)	0.88 (0.80 to 0.96)	0.97 (0.94 to 1.00)	0.02
**Full fat milk**	**<1 serving/month**	**<1 serving/week**	**1-3 servings/week**	**4-7 servings/week**	**>1 serving/day**		
No of participants/person years	7178/26 0187	111/8917	84/4682	147/6827	118/5353		
Age adjusted model	1	0.70 (0.58 to 0.84)	0.88 (0.71 to 1.10)	1.03 (0.87 to 1.21)	1.17 (0.97 to 1.40)	1.10 (1.00 to 1.20)	0.19
Multivariable model*	1	0.81 (0.67 to 0.98)	0.98 (0.79 to 1.22)	1.19 (1.01 to 1.41)	1.20 (1.00 to 1.45)	1.11 (1.02 to 1.21)	0.01
Fully adjusted model†	1	0.81 (0.67 to 0.98)	1.01 (0.81 to 1.26)	1.18 (1.00 to 1.40)	1.20 (0.99 to 1.44)	1.12 (1.02 to 1.22)	0.02

ASBs=artificially sweetened beverages; CI=confidence interval; SSBs=sugar sweetened beverages.

* Adjusted for age (continuous), duration of diabetes mellitus (years), sex (men or women), white ethnicity (yes or no), physical activity (<3.0, 3.0-8.9, 9.0-17.9, 18.0-26.9, ≥27.0 metabolic equivalents of task-hours/week), smoking status (never, former, current 1-14 cigarettes/day, current ≥15 cigarettes/day), alcohol consumption (0, 0.1-4.9, 5.0-14.9, ≥15.0 g/day), menopausal status and post-menopausal hormone use (pre-menopause, post-menopause (never, former, or current hormone use), or missing; Nurses’ Health Study only), family history of type 2 diabetes (yes or no) or myocardial infarction (yes or no), intake of total energy (continuous), and the modified Alternative Healthy Eating Index score (fourths). Individual beverage consumption was mutually adjusted.

† Further adjusted for history of hypertension (yes or no) or hypercholesterolemia (yes, no), use of antihypertensive (yes or no) or lipid lowering drug (yes or no), aspirin use (yes or no), diabetes drug use (oral drug only, insulin use, or others), and change in body mass index before to after diabetes diagnosis.

A total of 3447 (22.3%) adults with incident CVD were documented during 248 447 person years of follow-up. In the fully adjusted model, higher intake of SSBs was significantly associated with a higher risk of CVD; multivariable hazard ratio of CVD comparing the highest intake with lowest intake was 1.25 (95% confidence interval 1.03 to 1.51) (fig 1 and table 3). Conversely, increased consumption of coffee and low fat milk was inversely associated with CVD incidence: hazard ratio 0.82 (0.69 to 0.98) for coffee and 0.88 (0.78 to 1.00) for low fat milk.

**Table 3:** Hazard ratios for incident cardiovascular disease according to consumption of specific types of beverages among adults with type 2 diabetes

	Hazard ratio (95% CI) by consumption level					Every 1 serving/day	P value for trend	
**SSBs**	**<1 serving/month**	**<1 serving/week**	**1-3 servings/week**	**4-7 servings/week**	**>1 serving/day**			
No of participants/person years	2430/174 971	498/34 626	265/18 619	132/11 357	122/8874			
Age adjusted model	1	0.98 (0.89 to 1.08)	1.01 (0.89 to 1.14)	0.82 (0.69 to 0.98)	1.08 (0.90 to 1.30)	1.01 (0.94 to 1.09)		0.88
Multivariable model*	1	1.06 (0.96 to 1.17)	1.13 (0.99 to 1.29)	0.91 (0.76 to 1.09)	1.24 (1.03 to 1.51)	1.08 (1.00 to 1.16)		0.10
Fully adjusted model†	1	1.06 (0.96 to 1.17)	1.13 (0.99 to 1.28)	0.90 (0.75 to 1.08)	1.25 (1.03 to 1.51)	1.08 (1.00 to 1.16)		0.10
**ASBs**	**<1 serving/month**	**<3 servings/week**	**3-6 servings/week**	**1-2 servings/day**	**>2 servings/day**			
No of participants/person years	1372/103 280	621/43 031	657/47 303	421/29 713	376/25 121			
Age adjusted model	1	1.05 (0.95 to 1.15)	1.04 (0.94 to 1.14)	1.13 (1.01 to 1.27)	1.31 (1.16 to 1.47)	1.08 (1.04 to 1.11)		<0.001
Multivariable model*	1	1.04 (0.93 to 1.17)	0.97 (0.87 to 1.09)	1.06 (0.93 to 1.22)	1.04 (0.90 to 1.21)	1.01 (0.97 to 1.06)		0.58
Fully adjusted model†	1	1.03 (0.92 to 1.16)	0.97 (0.86 to 1.09)	1.05 (0.91 to 1.20)	1.02 (0.88 to 1.19)	1.01 (0.97 to 1.05)		0.78
**Fruit juice**	**<1 serving/month**	**<1 serving/week**	**1-3 servings/week**	**4-7 servings/week**	**>1 serving/day**			
No of participants/person years	1178/90 915	510/37 294	473/35 285	708/46 918	578/38 035			
Age adjusted model	1	1.00 (0.90 to 1.10)	1.02 (0.92 to 1.14)	1.01 (0.92 to 1.11)	0.98 (0.87 to 1.10)	1.01 (0.96 to 1.07)		0.97
Multivariable model*	1	1.06 (0.94 to 1.19)	1.07 (0.94 to 1.22)	1.03 (0.92 to 1.16)	1.09 (0.94 to 1.25)	1.02 (0.96 to 1.09)		0.38
Fully adjusted model†	1	1.05 (0.93 to 1.18)	1.06 (0.93 to 1.20)	1.02 (0.91 to 1.15)	1.08 (0.93 to 1.24)	1.02 (0.95 to 1.08)		0.48
**Coffee**	**<1 serving/month**	**<1 serving/day**	**1-2 servings/day**	**3-4 servings/day**	**>4 servings/day**			
No of participants/person years	1749/117 872	438/33 919	507/36 224	605/48 083	148/12 350			
Age adjusted model	1	0.87 (0.78 to 0.97)	0.91 (0.82 to 1.00)	0.87 (0.79 to 0.96)	0.90 (0.76 to 1.06)	0.97 (0.94 to 1.00)		0.01
Multivariable model*	1	0.91 (0.82 to 1.02)	0.97 (0.87 to 1.07)	0.89 (0.81 to 0.98)	0.80 (0.67 to 0.96)	0.96 (0.93 to 0.99)		0.004
Fully adjusted model†	1	0.90 (0.81 to 1.00)	0.95 (0.86 to 1.05)	0.89 (0.81 to 0.98)	0.82 (0.69 to 0.98)	0.96 (0.94 to 0.99)		0.006
**Tea**	**<1 serving/month**	**<3 servings/week**	**3-6 servings/week**	**1-2 servings/day**	**>2 servings/day**			
No of participants/person years	1943/135 675	585/45 187	405/29 547	278/19 127	236/18 911			
Age adjusted model	1	0.91 (0.83 to 1.00)	0.98 (0.88 to 1.09)	1.01 (0.89 to 1.15)	0.93 (0.81 to 1.07)	1.01 (0.97 to 1.05)		0.58
Multivariable model*	1	0.99 (0.90 to 1.09)	1.08 (0.96 to 1.20)	1.09 (0.96 to 1.24)	0.96 (0.84 to 1.11)	1.01 (0.97 to 1.05)		0.99
Fully adjusted model†	1	0.98 (0.89 to 1.08)	1.06 (0.95 to 1.18)	1.07 (0.94 to 1.22)	0.96 (0.84 to 1.11)	1.01 (0.97 to 1.05)		0.95
**Plain water**	**<1 serving/day**	**1 serving/day**	**2-3 servings/day**	**4-5 servings/day**	**>5 servings/day**			
No of participants/person years	1298/88 004	262/21 915	761/59 357	668/47 427	458/31 745			
Age adjusted model	1	0.87 (0.76 to 1.00)	0.86 (0.79 to 0.95)	0.87 (0.79 to 0.96)	0.87 (0.78 to 0.97)	0.97 (0.96 to 0.99)		0.002
Multivariable model*	1	0.90 (0.78 to 1.03)	0.93 (0.84 to 1.03)	0.98 (0.89 to 1.09)	0.98 (0.87 to 1.10)	0.99 (0.98 to 1.01)		0.94
Fully adjusted model†	1	0.89 (0.77 to 1.02)	0.91 (0.82 to 1.00)	0.95 (0.86 to 1.05)	0.94 (0.84 to 1.06)	0.94 (0.90 to 0.98)		0.45
**Low fat milk**	**<1 serving/month**	**<3 servings/week**	**3-6 servings/week**	**1-2 servings/day**	**>2 servings/day**			
No of participants/person years	1275/88 826	349/28 287	722/53 292	666/46 836	435/31 205			
Age adjusted model	1	0.86 (0.76 to 0.97)	0.88 (0.80 to 0.96)	0.87 (0.79 to 0.96)	0.82 (0.73 to 0.92)	0.95 (0.92 to 0.99)		0.003
Multivariable model*	1	0.89 (0.79 to 1.01)	0.96 (0.86 to 1.06)	0.95 (0.85 to 1.05)	0.89 (0.79 to 1.01)	0.98 (0.94 to 1.02)		0.18
Fully adjusted model†	1	0.87 (0.76 to 0.99)	0.93 (0.84 to 1.03)	0.93 (0.83 to 1.03)	0.88 (0.78 to 1.00)	0.98 (0.94 to 1.02)		0.16
**Full fat milk**	**<1 serving/month**	**<1 serving/week**	**1-3 servings/week**	**4-7 servings/week**	**>1 serving/day**			
No of participants/person years	3138/224 780	99/8231	51/4232	89/6291	70/4915			
Age adjusted model	1	0.94 (0.77 to 1.15)	0.97 (0.73 to 1.28)	1.06 (0.86 to 1.32)	1.13 (0.88 to 1.43)	1.14 (1.02 to 1.28)		0.30
Multivariable model*	1	0.94 (0.77 to 1.15)	0.96 (0.72 to 1.27)	1.05 (0.85 to 1.31)	1.09 (0.85 to 1.39)	1.13 (1.00 to 1.27)		0.45
Fully adjusted model†	1	0.93 (0.76 to 1.14)	0.95 (0.72 to 1.25)	1.05 (0.85 to 1.31)	1.11 (0.87 to 1.42)	1.14 (1.01 to 1.28)		0.37

ASBs=artificially sweetened beverages; CI=confidence interval; SSBs=sugar sweetened beverages.

* Analyses were adjusted for age (continuous), duration of diabetes mellitus (years), sex (men or women), white ethnicity (yes or no), physical activity (<3.0, 3.0-8.9, 9.0-17.9, 18.0-26.9, ≥27.0 metabolic equivalents of task-hours/week), smoking status (never, former, current 1-14 cigarettes/day, current ≥15 cigarettes/day), alcohol consumption (0, 0.1-4.9, 5.0-14.9, ≥15.0 g/day), menopausal status and post-menopausal hormone use (pre-menopause, post-menopause (never, former, or current hormone use), or missing; Nurses’ Health Study only), family history of type 2 diabetes (yes or no) or myocardial infarction (yes or no), intake of total energy (continuous), and the modified Alternative Healthy Eating Index score (fourths). Individual beverage consumption was mutually adjusted.

† Fully adjusted model, further adjusted for history of hypertension (yes or no) or hypercholesterolemia (yes or no), use of antihypertensive (yes or no) or lipid lowering drug (yes or no), aspirin use (yes or no), diabetes drug use (oral drug only, insulin use, or others), and change in body mass index before to after diabetes diagnosis.

**Fig 3:**
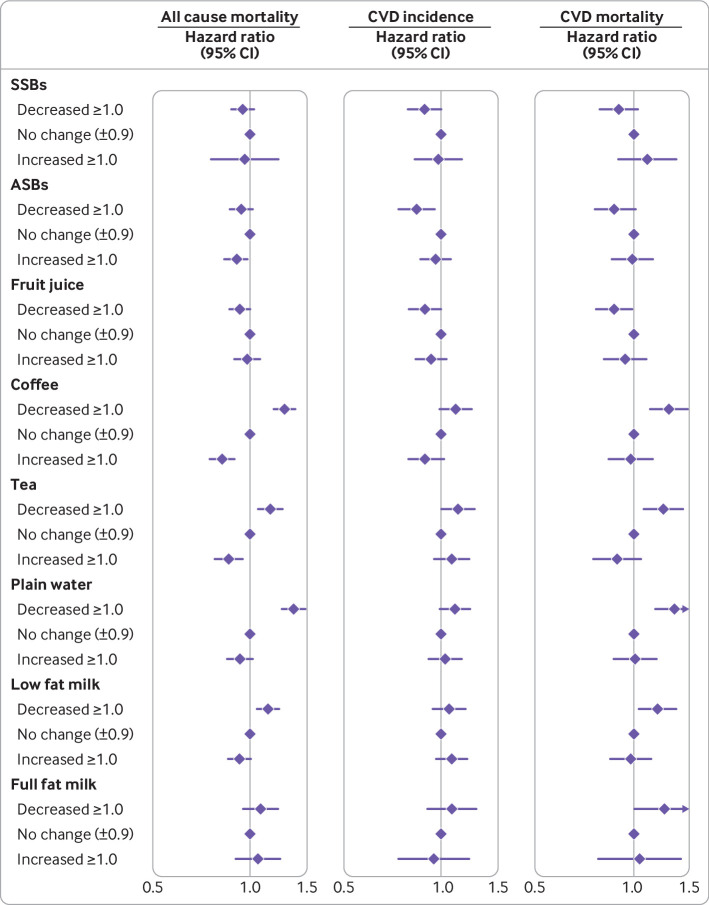
Hazard ratios (95% CIs) of all cause mortality and CVD incidence according to changes in consumption of specific types of beverages from before to after a diagnosis of type 2 diabetes. Hazard ratios were adjusted for age (continuous), duration of diabetes (years), sex (men or women), white (yes or no), physical activity (<3.0, 3.0-8.9, 9.0-17.9, 18.0-26.9, ≥27.0 metabolic equivalents of task-hours/week), smoking status (never, former, current 1-14 cigarettes/day, current ≥15 cigarettes/day), alcohol consumption (0, 0.1-4.9, 5.0-14.9, ≥15.0 g/day), menopausal status and post-menopausal hormone use (pre-menopause, post-menopause (never, former, or current hormone use), or missing; Nurses’ Health Study only), family history of type 2 diabetes (yes or no) or myocardial infarction (yes or no), intake of total energy (continuous), the modified Alternative Healthy Eating Index score (fourths), history of hypertension (yes or no) or hypercholesterolemia (yes or no), use of antihypertensive (yes or no) or lipid lowering drug (yes or no), aspirin use (yes or no), diabetes drug use (oral drug only, insulin use, or others), and change in body mass index before to after diabetes diagnosis. Individual beverage consumption was mutually adjusted. ASBs=artificially sweetened beverages; CI=confidence interval; CVD=cardiovascular disease; HR=hazard ratio; SSBs=sugar sweetened beverages

Increment in coffee consumption from before to after the diabetes diagnosis was also significantly associated with a lower risk of all cause mortality (fig 3 and supplementary table 3). Compared with participants whose coffee intake did not change, those who increased coffee consumption after a diabetes diagnosis had an associated 18% lower risk of all cause mortality. A similar pattern of associations in relation to all cause mortality was observed for tea and low fat milk. Associations between changes in intakes of these beverage and CVD incidence were similar but largely null owing to lower power.

In estimating the associations of substituting beverages for each other, when replacing one serving/day of SSBs, one serving/day of coffee was associated with an 18% (95% confidence interval 12% to 23%) lower risk of all cause mortality, tea with a 16% (9% to 22%) lower risk, plain water with a 16% (10% to 21%) lower risk, and low fat milk with a 12% (5% to 17%) lower risk (table 4). The corresponding substitution estimates for CVD mortality were 20%, 24%, 20%, and 19%. Replacing one serving/day of SSBs with ASBs was also significantly associated with 8% (95% 1% to 14%) and 15% (4% to 24%) lower all cause mortality and CVD mortality, respectively. Replacing one serving/day of ASBs with coffee, tea, or plain water was also associated with lower all cause mortality.

**Table 4:** Associations for replacing one serving/week of specific types of beverages on all cause mortality and cardiovascular disease incidence and mortality among adults with type 2 diabetes*

Beverage and substitutions	All cause mortality			CVD incidence			CVD mortality	
	Relative risk (95% CI)	P value		Relative risk (95% CI)	P value		Relative risk (95% CI)	P value
**SSBs**								
ASB	0.92 (0.86 to 0.99)	0.02		0.94 (0.86 to 1.04)	0.23		0.85 (0.76 to 0.96)	0.007
Fruit juice	0.97 (0.89 to 1.04)	0.22		0.96 (0.85 to 1.07)	0.46		0.92 (0.81 to 1.05)	0.21
Coffee	0.82 (0.77 to 0.88)	<0.001		0.91 (0.83 to 1.00)	0.05		0.80 (0.72 to 0.89)	<0.001
Tea	0.84 (0.78 to 0.91)	<0.001		0.93 (0.84 to 1.03)	0.18		0.76 (0.66 to 0.86)	<0.001
Plain water	0.84 (0.79 to 0.90)	<0.001		0.94 (0.86 to 1.03)	0.16		0.80 (0.72 to 0.89)	<0.001
Low fat milk	0.88 (0.83 to 0.95)	0.001		0.94 (0.85 to 1.04)	0.23		0.81 (0.72 to 0.91)	<0.001
Full fat milk	1.00 (0.89 to 1.13)	0.97		0.99 (0.82 to 1.20)	0.95		0.86 (0.69 to 1.07)	0.17
**ASBs**								
Fruit juice	1.05 (0.99 to 1.11)	0.11		1.01 (0.94 to 1.10)	0.73		1.08 (0.98 to 1.18)	0.13
Coffee	0.89 (0.85 to 0.93)	<0.001		0.96 (0.91 to 1.02)	0.19		0.94 (0.87 to 1.01)	0.08
Tea	0.91 (0.86 to 0.96)	<0.001		0.99 (0.93 to 1.06)	0.73		0.88 (0.80 to 0.98)	0.02
Plain water	0.91 (0.88 to 0.95)	<0.001		0.99 (0.95 to 1.04)	0.74		0.93 (0.88 to 0.99)	0.03
Low fat milk	0.96 (0.91 to 1.00)	0.07		0.99 (0.93 to 1.06)	0.87		0.95 (0.87 to 1.03)	0.22
Full fat milk	1.08 (0.97 to 1.20)	0.16		1.05 (0.89 to 1.24)	0.56		1.00 (0.82 to 1.23)	0.97
**Fruit juice**								
ASB	0.96 (0.90 to 1.01)	0.11		0.99 (0.91 to 1.07)	0.73		0.93 (0.84 to 1.02)	0.13
Coffee	0.85 (0.81 to 0.90)	<0.001		0.95 (0.88 to 1.03)	0.20		0.87 (0.79 to 0.95)	0.002
Tea	0.87 (0.82 to 0.93)	<0.001		0.97 (0.89 to 1.06)	0.55		0.82 (0.73 to 0.92)	<0.001
Plain water	0.87 (0.83 to 0.92)	<0.001		0.98 (0.91 to 1.05)	0.55		0.87 (0.80 to 0.94)	<0.001
Low fat milk	0.91 (0.86 to 0.97)	0.003		0.98 (0.90 to 1.07)	0.67		0.93 (0.76 to 1.15)	0.51
Full fat milk	1.03 (0.92 to 1.15)	0.59		1.04 (0.87 to 1.24)	0.70		0.93 (0.76 to 1.14)	0.49
**Full fat milk**								
Coffee	0.82 (0.74 to 0.92)	<0.001		0.92 (0.77 to 1.09)	0.31		0.88 (0.71 to 1.09)	0.24
Tea	0.84 (0.76 to 0.94)	0.003		0.94 (0.79 to 1.12)	0.48		0.93 (0.76 to 1.13)	0.48
Plain water	0.85 (0.76 to 0.94)	0.002		0.94 (0.80 to 1.11)	0.49		0.90 (0.72 to 1.13)	0.38
Low fat milk	0.89 (0.80 to 0.99)	0.02		0.95 (0.80 to 1.12)	0.51		0.94 (0.77 to 1.15)	0.57

ASBs=artificially sweetened beverages; CI=confidence interval; CVD=cardiovascular disease; SSBs=sugar sweetened beverages.

* Hazard ratios were adjusted for age (continuous), duration of diabetes mellitus (years), sex (men or women), white ethnicity (yes or no), physical activity (<3.0, 3.0-8.9, 9.0-17.9, 18.0-26.9, ≥27.0 metabolic equivalents of task-hours/week), smoking status (never, former, current 1-14 cigarettes/day, current ≥15 cigarettes/day), alcohol consumption (0, 0.1-4.9, 5.0-14.9, ≥15.0 g/day), menopausal status and post-menopausal hormone use (pre-menopause, post-menopause (never, former, or current hormone use), or missing; Nurses’ Health Study only), family history of type 2 diabetes (yes or no) or myocardial infarction (yes or no), intake of total energy (continuous), the modified Alternative Healthy Eating Index score (fourths), history of hypertension (yes or no) or hypercholesterolemia (yes or no), use of antihypertensive (yes or no) or lipid lowering drugs (yes or no), aspirin use (yes or no), diabetes drug use (oral drug only, insulin use, or others), and change in body mass index before to after diabetes diagnosis. Individual beverage consumption was mutually adjusted.

In sensitivity analyses, the associations between beverage consumptions and CVD risk and mortality remained robust when we excluded participants with prevalent diabetes at baseline, when we excluded deaths that occurred within four years after a diabetes diagnosis, and when we further adjusted for intakes of major dietary variables or beverage consumptions with a four year lag or eight year lag (see supplementary table 4). In sensitivity analyses adjusting for BMI before diabetes diagnosis instead of the change in BMI before to after diabetes diagnosis, the associations were largely similar to the results from primary analyses (see supplementary table 4). Estimates of associations were similar when further adjusting for socioeconomic status (see supplementary table 4). The J-shaped dose-response relationships for SSBs, ASBs, fruit juice, and full fat milk were also largely robust when we used beverage intake before the diabetes diagnosis instead of the cumulative average (see supplementary figure 2), or when we left out the first food frequency questionnaire data after the diabetes diagnosis (see supplementary figure 3). The results remained virtually unchanged when we restricted our analyses to never smokers (see supplementary table 4). Restricting to participants with asymptomatic type 2 diabetes at the time of diagnosis did not materially alter the associations (see supplementary table 4). In addition, further adjustment for the number of diabetes related symptoms or for HbA_1c_ levels did not appreciably change the results (see supplementary table 4).

## Discussion

In these two prospective cohorts of men and women with type 2 diabetes in the United States, we found that higher SSB intake was associated with higher all cause mortality and CVD incidence, whereas intakes of coffee, low fat milk, and plain water were inversely associated with CVD incidence and mortality. In addition, greater increase in coffee consumption from before to after a diabetes diagnosis was significantly associated with lower mortality. Replacing SSBs with ASBs was significantly associated with lower all cause and CVD mortality. In addition, replacing SSBs or ASBs with coffee, tea, low fat milk, or plain water was associated with lower all cause mortality and CVD mortality.

### Comparison with other studies and possible explanations

Abundant evidence links the intake of various beverages with cardiometabolic conditions and mortality in the general population. For example, meta-analyses of prospective cohort studies indicated that a higher consumption of SSBs was associated with weight gain, type 2 diabetes, and all cause mortality and CVD mortality.^10^ ^11^ ^29^ Several prospective cohort studies have suggested that ASB as a replacement for SSB consumption may increase the risk of obesity, metabolic diseases, and mortality in the general population.^30^ ^31^ However, the results from meta-analysis of randomized controlled trials showed that ASB consumption did not appear to influence blood glucose levels.^32^ In contrast, consumption of coffee, tea, and plain water was strongly associated with a lower risk of CVD, type 2 diabetes, cancer, and mortality.^9^ ^33^ ^34^ ^35^ In addition, evidence is accumulating that consumption of full fat milk is associated with a higher risk of all cause, CVD, and cancer mortality, whereas consumption of low fat milk was inversely associated with type 2 diabetes and risk of stroke.^7^ ^8^,^36^ In the European Prospective Investigation into Cancer and Nutrition-InterAct study, replacing SSBs for coffee or tea by 250 g/day was associated with a 21% or 22% lower incidence of type 2 diabetes across eight European populations.^37^

In contrast with the evidence from general populations, evidence from prospective epidemiological studies specifically for adults with type 2 diabetes is largely lacking. In the current analyses, we found a pattern of associations between individual beverages and mortality that largely mirrors that observed in general populations. In particular, we found that higher SSB intake was associated with higher CVD incidence and mortality among individuals with type 2 diabetes, whereas intakes of coffee, low fat milk, or plain water were inversely associated with CVD incidence and mortality. Our observed associations were also consistent with findings in some controlled trials that examined beverages in relation to cardiometabolic risk factors among adults with diabetes or those at higher risk of developing diabetes. In overweight participants who received four cups of coffee daily, 24 week intervention led to a significant reduction in fat mass.^38^ An eight week supplementation of coffee extract significantly reduced fasting insulin level and the homeostasis model assessment of insulin resistance in adults with metabolic syndrome.^39^ Daily supplementary intake of tea extract powder lowered the HbA_1c_ level and decreased serum C reactive protein levels in adults with diabetes.^14^ ^40^ A 12 week randomized trial suggested that supplementary intake of green tea extract may improve arterial stiffness in patients with type 2 diabetes.^15^ Overall, the consistency of evidence regarding individual beverage intake between populations with and without diabetes suggests that the altered metabolism among adults with diabetes does not play a critical role in modifying the associations of beverages and health outcomes.

Several biological mechanisms may explain the putative distinct associations of specific types of beverages with all cause mortality and CVD incidence among adults with diabetes. The positive association between SSB intake and adverse health outcomes may relate to the high fructose content in liquid form.^41^ ^42^ ^43^ ^44^ The added high fructose corn syrup and sucrose in SSBs may lead to weight gain, insulin resistance, and inflammation.^42^ A postprandial spike in blood glucose and insulin concentrations after SSB consumption may potentially lead to hyperinsulinemia, lipogenesis, and insulin resistance over time, which is particularly harmful for adults with diabetes.^43^ Also, calories in liquid from SSB consumption may decrease satiety and lead to incomplete compensatory reduction in energy intake, which consequently does not suppress consumption of solid foods.^44^ In addition to relatively higher contents of simple sugars in fruits juices, in comparison with whole fruits, fruit juice, on average, may have lower contents of some beneficial constituents, such as fiber, polyphenols, and other phytochemicals, which are partially lost during the juicing process.^45^ In contrast, coffee contains various beneficial bioactive constituents, such as chlorogenic acids, melanoidins, and trigonelline, which may reduce oxidative stress and inflammation.^46^ ^47^ ^48^ Regular consumption of coffee may significantly reduce systematic inflammation.^47^ ^48^ In particular, chlorogenic acid has also been shown to delay intestinal glucose uptake and inhibit hepatic gluconeogenesis.^49^ ^50^ ^51^ Tea is also a good source of polyphenols, especially catechins, which bear antioxidant and anti-inflammatory properties.^52^ Catechins could also modulate several key genes in triacylglycerol biosynthesis,^53^ protect pancreatic β cells,^54^ and improve the cellular redox state.^55^ The divergent associations between intake of full fat and low fat milk are in line with the evidence base that collectively suggests detrimental effects of saturated fat intake on blood lipids, systemic inflammation, and insulin sensitivity.^56^

In restricted cubic spline analysis, we observed a slight J-shaped relationship between consumption of some specific beverages (SSBs, ASBs, fruit juice, and full fat milk) and overall mortality. It is possible that underreporting or recent quitting of these beverages may potentially account for these observations, although the sensitivity analyses in which we modeled consumption levels before diabetes diagnosis or left out the first food frequency questionnaire assessment since diagnosis yielded similar findings. It would seem that more studies are warranted to replicate and further explore these important associations.

Our results suggested that replacing SSBs with ASBs was associated with a significantly lower risk of CVD incidence and mortality in adults with diabetes, after controlling for several covariates, including weight change from before to after the diabetes diagnosis, which is a strong predictor of adverse outcomes in our previous study.^27^ The observations are in accordance with the recommendations of the American Heart Association and American Diabetes Association, which suggest replacement of SSBs with non-caloric sweetened beverages for weight and glycemic control.^13^ Compared with SSBs that are high in sugars, ASBs contain sweeteners that provide limited or no energy content. The evidence is largely inconsistent about the effects of non-nutritive sweeteners and ASBs on glucose metabolism, gut microbiota, and cardiometabolic risk.^57^ ^58^ ^59^ In contrast with findings of a positive association of ASB consumption with weight gain, metabolic diseases, and mortality in prospective cohort studies, evidence from randomized controlled trials did not find a substantial impact of ASB consumption compared with SSB consumption on increasing blood glucose levels and inducing changes to gut microbiota.^31^ ^60^ ^61^ In addition, evidence from trials in humans had suggested that using non-caloric sweetened beverages as an intended substitute for beverages that contain sugar could result in a modest improvement in body weight and cardiometabolic risk factors, especially among people with obesity.^62^ ^63^ ^64^ The reason for inconsistent findings between cohort studies and trials is unclear, although the differences in study duration between trials and observational studies and confounding by weight loss attempts in observational studies may partially explain the discrepancies.

### Strengths and limitations of this study

The strengths of the current study include the large sample size, long duration of follow-up from two prospective cohort studies, high response rates during follow-up, and detailed and repeated assessments of dietary and lifestyle variables before and after a diabetes diagnosis.

Several potential limitations need to be considered as well. First, individual beverage consumption may be correlated with other dietary and lifestyle risk factors for CVD incidence and mortality among adults with diabetes. Given the observational nature of the study design, the possibility of residual confounding due to measurement errors of covariates (including severity of diabetes, glucose control, and dietary and lifestyle factors) and unmeasured confounding (including genetic susceptibility and psychosocial stress) cannot be completely ruled out, especially in this patient population—even though we controlled for a wide range of potential confounders. Therefore, our results should be interpreted with caution and warrant intervention studies to help establish causal relationships. Second, some measurement errors and misclassification are inevitable in estimates of food and nutrient intakes. The validation studies showed that our food frequency questionnaires have reasonable reproducibility and validity compared with diet record assessments.^19^ ^20^ ^65^ In particular, food frequency questionnaire assessments of beverage intake are, in general, strongly correlated with diet record assessments, probably because the consumption pattern is habitual. Use of repeated measurements of dietary intake to calculate cumulative averages is likely to help minimize random measurement errors and reflect long term dietary intakes. Third, self-reported diabetes diagnoses were ascertained using a validated supplementary questionnaire, although the possibility of some underdiagnosis of type 2 diabetes may exist. Previous studies have, however, clearly shown the validity of the supplementary questionnaire.^23^ ^24^ Moreover, the incidence rate of diabetes in our study populations was also comparable with that in other cohort studies.^66^ ^67^ ^68^ Because of the study participants’ professions and ready access to healthcare, underreporting of diabetes was expected to be less than that in the general population. A validation study assessing the prevalence of undiagnosed diabetes among a random sample of participants without type 2 diabetes in the Nurses’ Health Study showed a low false negative rate (0.5%) in this cohort.^69^ In addition, results did not significantly change when the analysis was restricted to adults with asymptomatic type 2 diabetes. Fourth, diabetes in participants in our study was diagnosed during an extended period since the 1980s. The risk profile of adults with diabetes might significantly change over time owing to better control of hypertension, blood lipids, and other risk factors in recent years; although similar results were found in analyses stratified by follow-up time. Fifth, our study did not have direct measurements of glycemic control and severity of diabetes, although the results did not materially change after further adjustment for duration of diabetes, use of diabetes drugs, diabetes symptoms, or HbA_1c_ levels. Lastly, the participants in our cohorts were predominantly white, US health professionals, which could potentially limit the generalizability of the findings to the general population. However, the range and frequency distribution of dietary macronutrient composition and diet quality for our cohort were similar to national estimates among US adults.^70^ ^71^ Although it is unlikely that the biologic mechanisms underlying these associations would differ substantially among different populations, further studies are warranted to replicate our findings in other populations.

### Conclusions

Findings from two large prospective cohort studies suggested that among adults with type 2 diabetes, coffee, plain water, and low fat milk are associated with lower risk of CVD or premature death, whereas the opposite association was found for excess intake of SSBs. Overall, these results provide additional evidence that emphasizes the importance of beverage choices in maintaining overall health among adults with diabetes.

**What is already known on this topic**

Depending on the content of sugar and other constituents, different types of beverages may have distinct health effects
The prevailing dietary recommendations are largely based on findings in the general US population
Evidence is limited among adults with type 2 diabetes, who have altered metabolism of energy and macronutrients

**What this study adds**

Among adults with type 2 diabetes, higher intake of sugar sweetened beverages (SSBs) was associated with higher all cause mortality and incidence of cardiovascular disease, whereas intakes of coffee, tea, plain water, or low fat milk were inversely associated with all cause mortality
Greater increase in coffee and tea consumption from before to after a diabetes diagnosis was significantly associated with lower all cause mortality
Replacing SSBs with coffee, tea, or plain water was statistically significantly associated with lower all cause mortality among adults with diabetes

## Data Availability

Data of the Nurses’ Health Study and Health Professionals Follow-Up Study can be shared through mechanisms detailed at https://www.nurseshealthstudy.org or https://www.hsph.harvard.edu/hpfs/.

## Web extra

Extra material supplied by authors

## Notes

Contributors: QS and LM participated in project conception and development of research methods. QS, FBH, EBR, and JEM obtained funding and provided oversight. LM, YH, DA, GL, VM, and QS analyzed data and performed analysis. LM and QS drafted the manuscript. All authors provided final approval of the version to be published. QS is the guarantor. The corresponding author attests that all listed authors meet authorship criteria and that no others meeting the criteria have been omitted.

The lead author (QS) affirms that the manuscript is an honest, accurate, and transparent account of the study being reported; that no important aspects of the study have been omitted; and that any discrepancies from the study as planned (and, if relevant, registered) have been explained.

Dissemination to participants and related patient and public communities: Findings from the current work will be disseminated through open access publication at *The BMJ*, our newsletters to study participants, the Health Professionals Follow-Up Study Twitter handle, and potential press releases.

Provenance and peer review: Not commissioned; externally peer reviewed.
