# Supplementary material for: Beverage consumption and mortality among adults with type 2 diabetes: prospective cohort study
Source: BMJ. 2023 Apr 19;381:e073406. doi: 10.1136/bmj-2022-073406 (PMC10114037; doi:10.1136/bmj-2022-073406)
Supplement: Supplementary file 1 — Supplementary information: Additional methods, figures 1-3, and tables 1-4 [file male073406.ww.pdf]

Beverage consumption and mortality among adults with type 2 diabetes: prospective cohort study

Supplementary materials

Online Appendix

Methods

Ascertainment of type 2 diabetes

Before 1998, the National Diabetes Data Group criteria were used to define type 2 diabetes.[1] A case of type 2 diabetes was confirmed if at least one of the following was reported on the supplementary questionnaire: 1) one or more diabetes-related symptoms (excessive thirst, polyuria, weight loss, hunger) plus fasting plasma glucose concentrations ≥7.8 mmol/L or random plasma glucose concentrations ≥11.1 mmol/L; 2) elevated plasma glucose concentrations on different occasions (fasting concentrations of ≥7.8 mmol/L, random plasma glucose concentrations of ≥11.1 mmol/L, and/or concentrations of ≥11.1 mmol/L during an oral glucose tolerance test in the absence of symptoms; or 3) treatment with hypoglycemic medication (insulin or oral hypoglycemic agent). For cases identified after 1998, the diagnosis criterion of the fasting plasma glucose threshold was lowered to 7.0 mmol/L by the American Diabetes Association (ADA) criteria.[2] After 2010, glycated hemoglobin HbA1c ≥6.5% was further included in the diagnosis criteria.[3]

Ascertainment of cardiovascular disease (CVD) and mortality

Incident CVD was defined as fatal and nonfatal coronary heart disease (including coronary artery bypass graft surgery and nonfatal myocardial infarction) and fatal and nonfatal stroke. When participants reported cardiovascular events on any biennial questionnaires, permission was requested to access their medical records. Study physicians who were blinded to the participant questionnaire data reviewed the medical records. Nonfatal myocardial infarction was confirmed according to the World Health Organization criteria, including typical symptoms plus either elevated cardiac enzyme concentrations or diagnostic electrocardiographic findings.[4] The diagnosis of coronary artery bypass graft surgery was based on self-report, for which the validity had been demonstrated.[5] Nonfatal stroke was defined based on the National Survey of Stroke criteria, requiring evidence of neurological deficits with sudden or rapid onset, which persisted for at least 24 h or until death.[6] Coronary heart disease and stroke events for which confirmatory information was obtained by interview or letter but no medical records were available were designated as "probable." Because the exclusion of probable CVD cases did not alter the results, we included both confirmed and probable cases of fatal and non-fatal coronary heart disease and

stroke in this analysis to maximize statistical power. Deaths were identified from reports by the next of kin or postal authorities or from searches of the National Death Index. Fatal coronary heart disease was defined if coronary heart disease was listed as the cause of death on the death certificate and the history of coronary heart disease was evident through reviewing hospital records, autopsy reports or other information. Similarly, fatal stroke was identified and confirmed by reviewing death certificates, hospital records, or autopsy records. The diagnostic codes of *the International Classification of Diseases, 9th Revision* (ICD-9), were used to classify deaths as due to CVD (ICD-9 codes 390-459), cancer (ICD-9 codes 140-208.32), or other causes.[7]

Assessment of covariates

In both cohorts, information on demographics, self-rated socioeconomic status (top 30%, median 40%, or bottom 30%, Nurses' Health Study only), partner's education (<high school, high school, or above college, Nurses' Health Study only), physical activity, smoking status, menopausal status and post-menopausal hormone use (Nurses' Health Study only), use of antihypertensive or lipid lowering drug, aspirin use, diabetes drug use (oral medication only, insulin use, or others), family history of myocardial infarction or type 2 diabetes, disease diagnoses (including hypertension, hypercholesterolemia, CVD, cancer, or other diseases) was collected at baseline and in biennial questionnaires. Alcohol and other dietary information were assessed and updated using validated food frequency questionnaires. Body mass index was calculated as weight in kilograms divided by the square of height in meters (kg/m^2). Physical activity was estimated as metabolic equivalents per week based on the average hours spent on various activities, weighted by the intensity level. Weight change was evaluated from the most recent biennial questionnaire before diabetes diagnosis to the first questionnaire after diagnosis. [8] Detailed descriptions on the validity and reproducibility of self-reported weight, physical activity, and alcohol consumption have been published elsewhere.[9-11] In addition, type 2 diabetes patients were also asked to report recent glycated hemoglobin HbA_{1c} levels (<7.0%, 7.0%-7.9%, 8.0%-9.9%, 10.0%-11.9%, and ≥12.0%) in supplementary questionnaires administered in 2000 and 2005 in the Nurses' Health Study and 2000, 2004, and 2008 in the Health Professionals Follow-Up Study.

References

1. National Diabetes Data Group. Classification and diagnosis of diabetes mellitus and other categories of glucose intolerance. *Diabetes* 1979;28:1039-57. doi: 10.2337/diab.28.12.1039.
2. American Diabetes Association. Standards of medical care in diabetes-2010. *Diabetes Care* 2010;33 Suppl 1:S11-61. doi: 10.2337/dc10-S011.
3. Hu FB, Manson JE, Stampfer MJ, et al. Diet, lifestyle, and the risk of type 2 diabetes mellitus in women. *N Engl J Med* 2001;345:790-7. doi: 10.1056/NEJMoa010492.
4. Rose GA. Cardiovascular Survey Methods. Geneva and Albany, NY: World Health Organization; WHO Publications Centre distributor, 1982.
5. Colditz GA, Martin P, Stampfer MJ, et al. Validation of questionnaire information on risk

factors and disease outcomes in a prospective cohort study of women. *Am J Epidemiol* 1986;123:894-900. doi: 10.1093/oxfordjournals.aje.a114319.

6. Walker AE, Robins M, Weinfeld FD. The National Survey of Stroke. Clinical findings. *Stroke* 1981;12(2 Pt 2 Suppl 1):I13-44.
7. Tobias DK, Pan A, Jackson CL, et al. Body-mass index and mortality among adults with incident type 2 diabetes. *N Engl J Med* 2014;370:233-44. doi: 10.1056/NEJMoa1304501.
8. Hu J, Hu Y, Hertzmark E, et al. Weight Change, Lifestyle, and Mortality in Patients With Type 2 Diabetes. *J Clin Endocrinol Metab* 2022;107(3):627-637. doi: 10.1210/clinem/dgab800.
9. Rimm EB, Stampfer MJ, Colditz GA, Chute CG, Litin LB, Willett WC. Validity of self-reported waist and hip circumferences in men and women. *Epidemiology* 1990;1:466-473. doi: 10.1097/00001648-199011000-00009.
10. Rimm EB, Giovannucci EL, Willett WC, et al. Prospective study of alcohol consumption and risk of coronary disease in men. *Lancet* 1991;338:464-8. doi: 10.1016/0140-6736(91)90542-w.
11. Wolf AM, Hunter DJ, Colditz GA, et al. Reproducibility and validity of a self-administered physical activity questionnaire. *Int J Epidemiol* 1994;23:991-9. doi: 10.1093/ije/23.5.991.

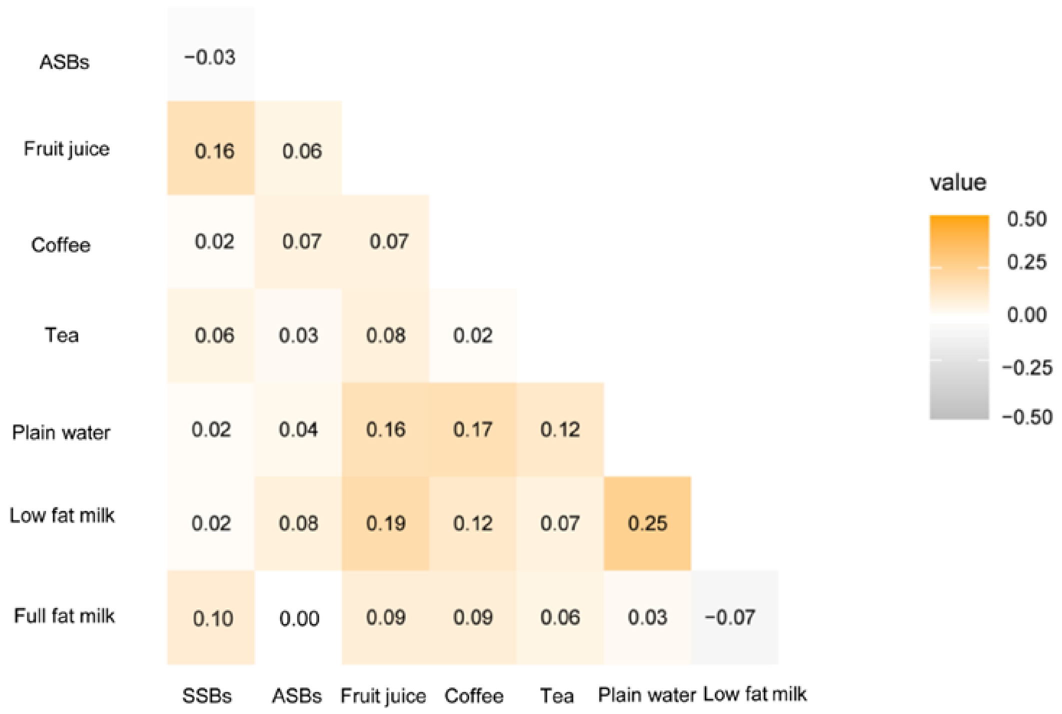

Supplementary Figure 1. Correlation coefficients between individual beverages

ASB: artificially sweetened beverages; SSB: sugar-sweetened beverages.

Supplementary Table 1. Hazard ratios for CVD mortality according to consumption of specific types of beverages among adults with type 2 diabetes. *

	Hazard ratio (95% CI) by consumption level					P value for trend
**SSBs**	**<1 serving/month**	**<1 serving/week**	**1-3 servings/week**	**4-7 servings/week**	**>1 serving/day**	
No of participants/person years	1738/200809	311/40299	169/21511	100/13109	79/10239	
Age adjusted model	1	0.89 (0.78 to 1.00)	0.96 (0.82 to 1.12)	0.99 (0.81 to 1.21)	1.14 (0.91 to 1.44)	0.26
Fully-adjusted model*	1	1.02 (0.90 to 1.15)	1.15 (0.98 to 1.35)	1.06 (0.86 to 1.31)	1.29 (1.02 to 1.63)	0.003
**ASBs**	**<1 serving/month**	**<3 servings/week**	**3-6 servings/week**	**1-2 servings/day**	**>2 servings/day**	
No of participants/person years	1167/117911	390/49777	414/54548	235/34472	191/29260	
Age adjusted model	1	0.81 (0.71 to 0.93)	0.89 (0.78 to 1.02)	0.93 (0.79 to 1.11)	0.90 (0.73 to 1.10)	0.002
Fully-adjusted model*	1	0.85 (0.74 to 0.98)	0.93 (0.81 to 1.07)	0.97 (0.82 to 1.16)	0.93 (0.75 to 1.14)	0.94
**Fruit juice**	**<1 serving/month**	**<1 serving/week**	**1-3 servings/week**	**4-7 servings/week**	**>1 serving/day**	
No of participants/person years	961/103402	287/42993	324/40754	452/54625	373/44193	
Age adjusted model	1	0.86 (0.76 to 0.97)	0.98 (0.86 to 1.11)	0.93 (0.83 to 1.04)	1.00 (0.87 to 1.15)	0.30
Fully-adjusted model*	1	0.91 (0.80 to 1.03)	1.03 (0.90 to 1.18)	0.98 (0.88 to 1.10)	1.07 (0.92 to 1.23)	0.66
**Coffee**	**<1 serving/month**	**<1 serving/day**	**1-2 servings/day**	**3-4 servings/day**	**>4 servings/day**	
No of participants/person years	1522/138144	271/39261	273/41830	270/53379	61/13354	
Age adjusted model	1	0.68 (0.59 to 0.77)	0.61 (0.54 to 0.70)	0.56 (0.49 to 0.64)	0.65 (0.51 to 0.85)	<0.001
Fully-adjusted model*	1	0.87 (0.76 to 1.00)	0.84 (0.73 to 0.96)	0.78 (0.68 to 0.90)	0.83 (0.64 to 1.08)	<0.001
**Tea**	**<1 serving/month**	**<3 servings/week**	**3-6 servings/week**	**1-2 servings/day**	**>2 servings/day**	
No of participants/person years	1706/158479	285/51426	187/33659	113/21457	106/20947	
Age adjusted model	1	0.63 (0.56 to 0.72)	0.60 (0.51 to 0.70)	0.63 (0.52 to 0.76)	0.68 (0.56 to 0.83)	<0.001
Fully-adjusted model*	1	0.85 (0.75 to 0.98)	0.83 (0.71 to 0.97)	0.84 (0.69 to 1.03)	0.89 (0.73 to 1.09)	0.03
**Plain water**	**<1 serving/day**	**1 serving/day**	**2-3 servings/day**	**3-5 servings/day**	**>5 servings/day**	
No of participants/person years	1315/103523	163/24751	439/67553	292/54188	188/35953	
Age adjusted model	1	0.67 (0.57 to 0.79)	0.58 (0.52 to 0.65)	0.48 (0.42 to 0.54)	0.53 (0.46 to 0.62)	<0.001
Fully-adjusted model*	1	0.88 (0.74 to 1.05)	0.82 (0.73 to 0.93)	0.70 (0.61 to 0.81)	0.77 (0.65 to 0.91)	<0.001
**Low fat milk**	**<1 serving/month**	**<3 servings/week**	**3-6 servings/week**	**1-2 servings/day**	**>2 servings/day**	
No of participants/person years	1287/103028	206/32359	341/61638	345/53761	218/35183	
Age adjusted model	1	0.63 (0.55 to 0.74)	0.50 (0.44 to 0.57)	0.57 (0.50 to 0.64)	0.56 (0.48 to 0.65)	<0.001
Fully-adjusted model*	1	0.92 (0.78 to 1.07)	0.76 (0.67 to 0.87)	0.85 (0.74 to 0.98)	0.84 (0.72 to 0.99)	0.004
**Full fat milk**	**<1 serving/month**	**<1 serving/week**	**1-3 servings/week**	**4-7 servings/week**	**>1 serving/day**	
No of participants/person years	2241/260187	39/8917	27/4682	51/6827	39/5353	
Age adjusted model	1	0.80 (0.58 to 1.09)	0.95 (0.65 to 1.39)	1.09 (0.82 to 1.44)	1.17 (0.85 to 1.61)	0.31
Fully-adjusted model*	1	0.91 (0.66 to 1.25)	1.07 (0.73 to 1.57)	1.28 (0.96 to 1.70)	1.25 (0.90 to 1.73)	0.24

ASB=artificially sweetened beverages; CI=confidence interval; CVD=cardiovascular disease; HR=hazard ratio; SSB=sugar-sweetened beverages.

* Analyses were adjusted for age (continuous), duration of diabetes mellitus (years), sex (men or women), white ethnicity (yes or no), physical activity (<3.0, 3.0-8.9, 9.0-17.9, 18.0-26.9, ≥27.0 metabolic equivalents-hours/week), smoking status (never, former, current 1-14 cigarettes/day, current ≥15 cigarettes/day), alcohol consumption (0, 0.1-4.9, 5.0-14.9, ≥15.0 g/day), menopausal status and post-menopausal hormone use (pre-menopause, post-menopause (never, former, or current hormone use), or missing; Nurses' Health Study only), family history of type 2 diabetes (yes or no) or myocardial infarction (yes or no), intake of total energy (continuous), the modified Alternative Healthy Eating Index score (fourths), history of hypertension (yes or no) or hypercholesterolemia (yes or no), use of antihypertensive (yes or no) or lipid lowering drug (yes or no), aspirin use (yes or no), diabetes drug use (oral drug only, insulin use, or others), and change in body mass index before to after diabetes diagnosis. Individual beverage consumption was mutually adjusted.

Supplementary Table 2. Hazard ratios for cancer mortality according to consumption of specific types of beverages among adults with type 2 diabetes. *

	Hazard ratio (95% CI) by consumption level					P value for trend
**SSBs**	**<1 serving/month**	**<1 serving/week**	**1-3 servings/week**	**4-7 servings/week**	**>1 serving/day**	
No of participants/person years	1038/200809	184/40299	113/21511	57/13109	41/10239	
Age adjusted model	1	0.88 (0.75 to 1.02)	1.06 (0.88 to 1.29)	0.92 (0.71 to 1.21)	0.98 (0.72 to 1.35)	0.82
Fully-adjusted model*	1	0.98 (0.84 to 1.16)	1.22 (1.00 to 1.49)	1.01 (0.77 to 1.33)	1.03 (0.75 to 1.42)	0.56
**ASBs**	**<1 serving/month**	**<3 servings/week**	**3-6 servings/week**	**1-2 servings/day**	**>2 servings/day**	
No of participants/person years	642/117911	247/49777	282/54548	132/34472	130/29260	
Age adjusted model	1	0.95 (0.80 to 1.12)	1.04 (0.88 to 1.22)	0.89 (0.72 to 1.11)	1.16 (0.93 to 1.44)	0.08
Fully-adjusted model*	1	0.96 (0.81 to 1.14)	1.06 (0.90 to 1.25)	0.90 (0.73 to 1.12)	1.18 (0.94 to 1.47)	0.15
**Fruit juice**	**<1 serving/month**	**<1 serving/week**	**1-3 servings/week**	**4-7 servings/week**	**>1 serving/day**	
No of participants/person years	553/103402	210/42993	183/40754	263/54625	224/44193	
Age adjusted model	1	0.98 (0.84 to 1.14)	0.95 (0.81 to 1.13)	0.98 (0.85 to 1.13)	0.88 (0.73 to 1.06)	0.32
Fully-adjusted model*	1	1.02 (0.87 to 1.19)	0.99 (0.83 to 1.17)	1.03 (0.89 to 1.20)	0.93 (0.77 to 1.13)	0.29
**Coffee**	**<1 serving/month**	**<1 serving/day**	**1-2 servings/day**	**3-4 servings/day**	**>4 servings/day**	
No of participants/person years	808/138144	178/39261	190/41830	212/53379	45/13354	
Age adjusted model	1	0.80 (0.68 to 0.94)	0.79 (0.68 to 0.93)	0.78 (0.67 to 0.90)	0.81 (0.60 to 1.10)	0.002
Fully-adjusted model*	1	0.96 (0.81 to 1.14)	0.96 (0.81 to 1.13)	0.88 (0.75 to 1.03)	0.80 (0.58 to 1.09)	0.007
**Tea**	**<1 serving/month**	**<3 servings/week**	**3-6 servings/week**	**1-2 servings/day**	**>2 servings/day**	
No of participants/person years	939/158479	199/51426	136/33659	89/21457	70/20947	
Age adjusted model	1	0.74 (0.63 to 0.86)	0.76 (0.64 to 0.91)	0.84 (0.67 to 1.04)	0.77 (0.60 to 0.98)	0.03
Fully-adjusted model*	1	0.91 (0.77 to 1.07)	0.97 (0.80 to 1.16)	1.04 (0.83 to 1.31)	0.91 (0.71 to 1.17)	0.27
**Plain water**	**<1 serving/day**	**1 serving/day**	**2-3 servings/day**	**3-5 servings/day**	**>5 servings/day**	
No of participants/person years	681/103523	106/24751	285/67553	219/54188	142/35953	
Age adjusted model	1	0.77 (0.63 to 0.95)	0.70 (0.60 to 0.80)	0.65 (0.55 to 0.76)	0.70 (0.58 to 0.84)	<0.001
Fully-adjusted model*	1	0.96 (0.77 to 1.18)	0.91 (0.78 to 1.06)	0.87 (0.74 to 1.03)	0.94 (0.77 to 1.14)	0.006
**Low fat milk**	**<1 serving/month**	**<3 servings/week**	**3-6 servings/week**	**1-2 servings/day**	**>2 servings/day**	
No of participants/person years	657/103028	122/32359	260/61638	236/53761	158/35183	
Age adjusted model	1	0.69 (0.56 to 0.84)	0.72 (0.62 to 0.83)	0.75 (0.65 to 0.88)	0.77 (0.65 to 0.93)	0.02
Fully-adjusted model*	1	0.91 (0.74 to 1.11)	1.00 (0.85 to 1.17)	1.06 (0.90 to 1.26)	1.10 (0.91 to 1.34)	0.49
**Full fat milk**	**<1 serving/month**	**<1 serving/week**	**1-3 servings/week**	**4-7 servings/week**	**>1 serving/day**	
No of participants/person years	1326/260187	23/8917	27/4682	28/6827	29/5353	
Age adjusted model	1	0.72 (0.47 to 1.08)	1.53 (1.04 to 2.24)	1.02 (0.70 to 1.49)	1.51 (1.04 to 2.19)	0.03
Fully-adjusted model*	1	0.76 (0.50 to 1.15)	1.60 (1.08 to 2.35)	1.07 (0.73 to 1.57)	1.54 (1.05 to 2.25)	0.11

ASB=artificially sweetened beverages; CI=confidence interval; HR=hazard ratio; SSB=sugar-sweetened beverages.

[*] Analyses were adjusted for age (continuous), duration of diabetes mellitus (years), sex (men or women), white ethnicity (yes or no), physical activity (<3.0, 3.0-8.9, 9.0-17.9, 18.0-26.9, ≥27.0 metabolic equivalents-hours/week), smoking status (never, former, current 1-14 cigarettes/day, current ≥15 cigarettes/day), alcohol consumption (0, 0.1-4.9, 5.0-14.9, ≥15.0 g/day), menopausal status and post-menopausal hormone use (pre-menopause, post-menopause (never, former, or current hormone use), or missing; Nurses' Health Study only), family history of type 2 diabetes (yes or no) or myocardial infarction (yes or no), intake of total energy (continuous), the modified Alternative Healthy Eating Index score (fourths), history of hypertension (yes or no) or hypercholesterolemia (yes or no), use of antihypertensive (yes or no) or lipid lowering drug (yes or no), aspirin use (yes or no), diabetes drug use (oral drug only, insulin use, or others), and change in body mass index before to after diabetes diagnosis. Individual beverage consumption was mutually adjusted.

Supplementary Table 3. Hazard ratios of all cause mortality, cardiovascular disease incidence, and cardiovascular disease mortality according to changes in consumption of specific types of beverages from before to after a diagnosis of type 2 diabetes.*

	**Change in beverage consumption, servings/day**			**P value**
	**Decreased ≥1.0**	**No change (±0.9)**	**Increased ≥1.0**	**for trend**
**All cause mortality**				
SSBs	0.95 (0.89 to 1.02)	1	1.05 (0.95 to 1.17)	0.04
ASBs	0.94 (0.87 to 1.01)	1	0.91 (0.84 to 0.97)	0.43
Fruit juice	0.93 (0.87 to 0.99)	1	0.98 (0.91 to 1.06)	0.10
Coffee	1.28 (1.19 to 1.37)	1	0.82 (0.76 to 0.89)	<0.001
Tea	1.16 (1.08 to 1.25)	1	0.86 (0.79 to 0.94)	<0.001
Plain water	1.37 (1.28 to 1.46)	1	0.93 (0.86 to 1.01)	<0.001
Low fat milk	1.14 (1.06 to 1.22)	1	0.93 (0.87 to 1.00)	<0.001
Full fat milk	1.08 (0.97 to 1.21)	1	1.06 (0.91 to 1.23)	0.52
**Cardiovascular disease incidence**				
SSBs	0.89 (0.80 to 0.99)	1	0.98 (0.84 to 1.15)	0.09
ASBs	0.84 (0.75 to 0.95)	1	0.96 (0.87 to 1.06)	0.08
Fruit juice	0.89 (0.80 to 0.99)	1	0.93 (0.83 to 1.03)	0.39
Coffee	1.11 (1.00 to 1.23)	1	0.89 (0.80 to 1.01)	0.002
Tea	1.13 (1.01 to 1.26)	1	1.08 (0.96 to 1.21)	0.30
Plain water	1.10 (1.00 to 1.22)	1	1.03 (0.93 to 1.15)	0.21
Low fat milk	1.06 (0.95 to 1.18)	1	1.08 (0.97 to 1.19)	0.72
Full fat milk	1.08 (0.91 to 1.27)	1	0.95 (0.74 to 1.21)	0.33
**Cardiovascular disease mortality**				
SSBs	0.90 (0.79 to 1.02)	1	1.10 (0.91 to 1.33)	0.04
ASBs	0.87 (0.77 to 1.00)	1	0.99 (0.87 to 1.13)	0.12
Fruit juice	0.87 (0.77 to 0.98)	1	0.94 (0.82 to 1.08)	0.15
Coffee	1.28 (1.13 to 1.45)	1	0.98 (0.85 to 1.13)	<0.001
Tea	1.23 (1.09 to 1.40)	1	0.89 (0.76 to 1.04)	<0.001
Plain water	1.33 (1.18 to 1.50)	1	1.01 (0.87 to 1.16)	<0.001
Low fat milk	1.18 (1.04 to 1.33)	1	0.98 (0.86 to 1.12)	0.01
Full fat milk	1.24 (1.01 to 1.51)	1	1.04 (0.79 to 1.38)	0.12

ASB=artificially sweetened beverages; CI=confidence interval; CVD=cardiovascular disease; HR=hazard ratio; SSB=sugar-sweetened beverages.

* Analyses were adjusted for age (continuous), duration of diabetes mellitus (years), sex (men or women), white ethnicity (yes or no), physical activity (<3.0, 3.0-8.9, 9.0-17.9, 18.0-26.9, ≥27.0 metabolic equivalents-hours/week), smoking status (never, former, current 1-14 cigarettes/day, current ≥15 cigarettes/day), alcohol consumption (0, 0.1-4.9, 5.0-14.9, ≥15.0 g/day), menopausal status and post-menopausal hormone use (pre-menopause, post-menopause (never, former, or current hormone use), or missing; Nurses' Health Study only), family history of type 2 diabetes (yes or no) or myocardial infarction (yes or no), intake of total energy (continuous), the modified Alternative Healthy Eating Index score (fourths), history of hypertension (yes or no) or hypercholesterolemia (yes or no), use of antihypertensive (yes or no) or lipid lowering drug (yes or no), aspirin use (yes or no), diabetes drug use (oral drug only, insulin use, or others), and change in body mass index before to after diabetes diagnosis. Individual beverage consumption was mutually adjusted.

Supplementary Table 4. Sensitivity analyses for the association between consumption of specific types of beverages and all cause mortality *

	Hazard ratio (95% CI) by consumption level					P value for trend
**SSBs**	**<1 serving/month**	**<1 serving/week**	**1-3 servings/week**	**4-7 servings/week**	**>1 serving/day**	
Excluding those with prevalent diabetes at baseline	1	0.95 (0.87 to 1.03)	1.00 (0.89 to 1.12)	1.06 (0.92 to 1.22)	1.36 (1.17 to 1.57)	<0.001
Excluding deaths that occurred within four years after the diabetes diagnosis	1	0.91 (0.84 to 1.00)	1.01 (0.90 to 1.13)	1.04 (0.90 to 1.20)	1.27 (1.09 to 1.47)	0.002
Using a four year lag period	1	0.92 (0.84 to 1.01)	1.04 (0.92 to 1.17)	1.03 (0.89 to 1.21)	1.23 (1.04 to 1.45)	<0.001
Using an eight year lag period	1	0.94 (0.86 to 1.04)	1.01 (0.89 to 1.15)	1.01 (0.86 to 1.19)	1.14 (0.96 to 1.36)	0.13
Adjustment for the BMI before the diabetes diagnosis	1	0.97 (0.91 to 1.05)	1.01 (0.92 to 1.11)	1.02 (0.91 to 1.15)	1.19 (1.04 to 1.37)	0.01
Using beverage intake assessed before the diabetes diagnosis	1	0.95 (0.88 to 1.02)	1.03 (0.94 to 1.12)	1.01 (0.90 to 1.12)	1.18 (1.04 to 1.33)	0.01
Skipping the first food frequency questionnaire after the diagnosis	1	0.93 (0.87 to 0.99)	0.90 (0.83 to 0.97)	0.99 (0.87 to 1.11)	1.07 (0.94 to 1.23)	0.29
Further adjusting for measures of socioeconomic status	1	0.97 (0.89 to 1.05)	1.04 (0.93 to 1.17)	1.08 (0.94 to 1.24)	1.28 (1.09 to 1.49)	0.001
Excluding current and former smokers	1	1.02 (0.91 to 1.15)	1.06 (0.91 to 1.23)	1.07 (0.89 to 1.29)	1.29 (1.07 to 1.57)	0.02
Asymptomatic type 2 diabetes cases only	1	0.99 (0.92 to 1.07)	1.02 (0.92 to 1.13)	1.03 (0.90 to 1.16)	1.21 (1.05 to 1.40)	0.009
Further adjusting for the number of diabetes related symptoms	1	0.74 (0.70 to 0.79)	0.83 (0.77 to 0.88)	0.89 (0.82 to 0.96)	1.16 (1.06 to 1.26)	0.02
Further adjusting for the self-reported levels of glycated hemoglobin HbA_{1c}	1	1.11 (0.98 to 1.26)	1.03 (0.86 to 1.23)	1.15 (0.92 to 1.45)	1.15 (0.90 to 1.47)	0.14
**ASBs**	**<1 serving/month**	**<3 servings/week**	**3-6 servings/week**	**1-2 servings/day**	**>2 servings/day**	
Excluding those with prevalent diabetes at baseline	1	0.84 (0.78 to 0.91)	0.90 (0.83 to 0.97)	0.88 (0.80 to 0.97)	0.95 (0.85 to 1.06)	0.34
Excluding deaths that occurred within four years after the diabetes diagnosis	1	0.85 (0.78 to 0.91)	0.91 (0.84 to 0.98)	0.89 (0.81 to 0.99)	0.97 (0.86 to 1.08)	0.61
Using a four year lag period	1	0.84 (0.77 to 0.91)	0.89 (0.81 to 0.97)	0.87 (0.78 to 0.98)	0.92 (0.80 to 1.06)	0.19
Using an eight year lag period	1	0.87 (0.79 to 0.97)	0.94 (0.84 to 1.04)	0.95 (0.82 to 1.09)	0.99 (0.84 to 1.17)	>0.99
Adjustment for the BMI before the diabetes diagnosis	1	0.83 (0.77 to 0.89)	0.88 (0.82 to 0.95)	0.86 (0.78 to 0.95)	0.96 (0.86 to 1.08)	0.41
Using beverage intake assessed before the diabetes diagnosis	1	0.82 (0.76 to 0.88)	0.88 (0.81 to 0.95)	0.83 (0.75 to 0.91)	0.94 (0.85 to 1.05)	0.10
Skipping the first food frequency questionnaire after the diagnosis	1	0.82 (0.77 to 0.88)	0.90 (0.84 to 0.97)	0.94 (0.85 to 1.03)	0.97 (0.87 to 1.09)	0.73
Further adjusting for measures of socioeconomic status	1	0.83 (0.76 to 0.91)	0.89 (0.81 to 0.97)	0.83 (0.74 to 0.94)	0.97 (0.85 to 1.10)	0.51
Excluding current and former smokers	1	1.08 (0.94 to 1.24)	0.94 (0.81 to 1.09)	0.98 (0.81 to 1.18)	1.17 (0.94 to 1.46)	0.35
Asymptomatic type 2 diabetes cases only	1	0.82 (0.76 to 0.89)	0.89 (0.82 to 0.96)	0.83 (0.75 to 0.92)	0.97 (0.86 to 1.09)	0.45
Further adjusting for the number of diabetes related symptoms	1	0.82 (0.77 to 0.89)	0.87 (0.81 to 0.94)	0.85 (0.77 to 0.94)	0.96 (0.86 to 1.07)	0.32
Further adjusting for the self-reported levels of glycated hemoglobin HbA_{1c}	1	0.78 (0.68 to 0.88)	0.96 (0.85 to 1.08)	0.96 (0.82 to 1.12)	1.10 (0.92 to 1.31)	0.06
**Fruit juice**	**<1 serving/month**	**<1 serving/week**	**1-3 servings/week**	**4-7 servings/week**	**>1 serving/day**	
Excluding those with prevalent diabetes at baseline	1	0.82 (0.76 to 0.88)	0.86 (0.80 to 0.93)	0.88 (0.83 to 0.94)	0.95 (0.88 to 1.02)	0.22
Excluding deaths that occurred within four years after the diabetes diagnosis	1	0.86 (0.80 to 0.93)	0.93 (0.86 to 1.00)	0.94 (0.88 to 1.01)	1.00 (0.93 to 1.08)	0.63
Using a four year lag period	1	0.84 (0.78 to 0.91)	0.89 (0.82 to 0.96)	0.89 (0.83 to 0.95)	0.96 (0.89 to 1.03)	0.33
Using an eight year lag period	1	0.86 (0.80 to 0.94)	0.95 (0.88 to 1.04)	0.91 (0.84 to 0.98)	1.01 (0.93 to 1.09)	0.88

Adjustment for the BMI before the diabetes diagnosis	1	0.87 (0.81 to 0.93)	0.92 (0.86 to 1.00)	0.88 (0.83 to 0.94)	0.98 (0.90 to 1.06)	0.12
Using beverage intake assessed before the diabetes diagnosis	1	0.95 (0.89 to 1.02)	0.97 (0.90 to 1.05)	0.94 (0.88 to 1.00)	0.95 (0.88 to 1.03)	0.14
Skipping the first food frequency questionnaire after the diagnosis	1	0.87 (0.81 to 0.94)	0.91 (0.85 to 0.97)	0.99 (0.92 to 1.06)	0.95 (0.88 to 1.02)	0.74
Further adjusting for measures of socioeconomic status	1	0.89 (0.82 to 0.96)	0.94 (0.86 to 1.03)	0.89 (0.82 to 0.96)	0.99 (0.89 to 1.09)	0.16
Excluding current and former smokers	1	0.88 (0.80 to 0.97)	0.87 (0.78 to 0.97)	0.86 (0.79 to 0.95)	0.98 (0.87 to 1.09)	0.13
Asymptomatic type 2 diabetes cases only	1	0.86 (0.80 to 0.92)	0.92 (0.85 to 0.99)	0.88 (0.82 to 0.94)	0.98 (0.89 to 1.06)	0.10
Further adjusting for the number of diabetes related symptoms	1	0.87 (0.81 to 0.93)	0.92 (0.86 to 1.00)	0.89 (0.83 to 0.95)	0.98 (0.90 to 1.06)	0.13
Further adjusting for the self-reported levels of glycated hemoglobin HbA_{1c}	1	1.08 (0.73 to 1.60)	0.96 (0.69 to 1.34)	0.88 (0.59 to 1.30)	0.88 (0.62 to 1.26)	0.40
**Coffee**	**<1 serving/month**	**<1 serving/day**	**1-2 servings/day**	**3-4 servings/day**	**>4 servings/day**	
Excluding those with prevalent diabetes at baseline	1	0.89 (0.81 to 0.97)	0.83 (0.76 to 0.90)	0.72 (0.66 to 0.79)	0.67 (0.55 to 0.81)	<0.001
Excluding deaths that occurred within four years after the diabetes diagnosis	1	0.89 (0.82 to 0.98)	0.81 (0.74 to 0.89)	0.69 (0.63 to 0.76)	0.64 (0.53 to 0.78)	<0.001
Using a four year lag period	1	0.88 (0.80 to 0.97)	0.83 (0.76 to 0.91)	0.71 (0.65 to 0.78)	0.64 (0.51 to 0.78)	<0.001
Using an eight year lag period	1	0.87 (0.79 to 0.96)	0.80 (0.73 to 0.89)	0.70 (0.64 to 0.78)	0.67 (0.54 to 0.83)	<0.001
Adjustment for the BMI before the diabetes diagnosis	1	0.84 (0.78 to 0.91)	0.83 (0.77 to 0.90)	0.72 (0.67 to 0.78)	0.74 (0.63 to 0.86)	<0.001
Using beverage intake assessed before the diabetes diagnosis	1	0.89 (0.81 to 0.97)	0.83 (0.76 to 0.90)	0.72 (0.66 to 0.79)	0.67 (0.55 to 0.81)	<0.001
Skipping the first food frequency questionnaire after the diagnosis	1	1.01 (0.96 to 1.08)	0.88 (0.83 to 0.95)	0.81 (0.75 to 0.88)	0.79 (0.64 to 0.97)	<0.001
Further adjusting for measures of socioeconomic status	1	0.88 (0.81 to 0.97)	0.83 (0.76 to 0.91)	0.70 (0.64 to 0.77)	0.70 (0.57 to 0.85)	<0.001
Excluding current and former smokers	1	0.83 (0.71 to 0.96)	0.85 (0.74 to 0.99)	0.67 (0.57 to 0.78)	0.66 (0.43 to 1.00)	<0.001
Asymptomatic type 2 diabetes cases only	1	0.86 (0.79 to 0.93)	0.82 (0.76 to 0.89)	0.72 (0.66 to 0.78)	0.71 (0.60 to 0.84)	<0.001
Further adjusting for the number of diabetes related symptoms	1	0.84 (0.78 to 0.91)	0.83 (0.77 to 0.90)	0.72 (0.67 to 0.78)	0.74 (0.63 to 0.86)	<0.001
Further adjusting for the self-reported levels of glycated hemoglobin HbA_{1c}	1	0.90 (0.79 to 1.02)	0.88 (0.77 to 1.00)	0.72 (0.63 to 0.82)	0.76 (0.58 to 0.99)	<0.001
**Plain water**	**<1 serving/day**	**1 serving/day**	**2-3 servings/day**	**3-5 servings/day**	**>5 servings/day**	
Excluding those with prevalent diabetes at baseline	1	0.78 (0.69 to 0.88)	0.79 (0.73 to 0.85)	0.73 (0.67 to 0.80)	0.75 (0.68 to 0.84)	<0.001
Excluding deaths that occurred within four years after the diabetes diagnosis	1	0.79 (0.70 to 0.89)	0.78 (0.72 to 0.84)	0.74 (0.68 to 0.81)	0.73 (0.66 to 0.82)	<0.001
Using a four year lag period	1	0.79 (0.69 to 0.90)	0.81 (0.74 to 0.88)	0.76 (0.69 to 0.83)	0.75 (0.67 to 0.84)	<0.001
Using an eight year lag period	1	0.78 (0.68 to 0.90)	0.82 (0.75 to 0.90)	0.76 (0.69 to 0.84)	0.79 (0.70 to 0.89)	<0.001
Adjustment for the BMI before the diabetes diagnosis	1	0.81 (0.76 to 0.88)	0.86 (0.79 to 0.94)	0.84 (0.76 to 0.94)	0.82 (0.73 to 0.92)	<0.001
Using beverage intake assessed before the diabetes diagnosis	1	0.87 (0.79 to 0.96)	0.86 (0.80 to 0.92)	0.80 (0.75 to 0.87)	0.85 (0.78 to 0.93)	<0.001
Skipping the first food frequency questionnaire after the diagnosis	1	0.72 (0.58 to 0.89)	0.98 (0.93 to 1.04)	0.87 (0.81 to 0.94)	0.82 (0.74 to 0.90)	<0.001
Further adjusting for measures of socioeconomic status	1	0.82 (0.73 to 0.93)	0.79 (0.73 to 0.86)	0.74 (0.68 to 0.81)	0.74 (0.67 to 0.83)	<0.001
Excluding current and former smokers	1	0.73 (0.59 to 0.90)	0.78 (0.68 to 0.88)	0.71 (0.62 to 0.82)	0.68 (0.58 to 0.81)	<0.001
Asymptomatic type 2 diabetes cases only	1	0.84 (0.75 to 0.93)	0.77 (0.72 to 0.83)	0.73 (0.68 to 0.79)	0.75 (0.68 to 0.83)	<0.001
Further adjusting for the number of diabetes related symptoms	1	0.84 (0.76 to 0.92)	0.79 (0.74 to 0.85)	0.73 (0.68 to 0.79)	0.77 (0.70 to 0.85)	<0.001
Further adjusting for the self-reported levels of glycated hemoglobin HbA_{1c}	1	0.86 (0.73 to 1.01)	0.89 (0.80 to 1.00)	0.81 (0.71 to 0.92)	0.84 (0.71 to 0.99)	0.002
**Low fat milk**	**<1 serving/month**	**<3 servings/week**	**3-6 servings/week**	**1-2 servings/day**	**>2 servings/day**	

Excluding those with prevalent diabetes at baseline	1	0.90 (0.81 to 0.99)	0.89 (0.82 to 0.97)	0.90 (0.83 to 0.99)	0.88 (0.79 to 0.98)	0.08
Excluding deaths that occurred within four years after the diabetes diagnosis	1	0.88 (0.79 to 0.98)	0.87 (0.80 to 0.95)	0.89 (0.81 to 0.97)	0.86 (0.77 to 0.95)	0.02
Using a four year lag period	1	0.91 (0.81 to 1.01)	0.87 (0.79 to 0.95)	0.95 (0.87 to 1.05)	0.88 (0.78 to 0.99)	0.16
Using an eight year lag period	1	0.86 (0.77 to 0.97)	0.85 (0.77 to 0.93)	0.88 (0.80 to 0.97)	0.84 (0.75 to 0.95)	0.03
Adjustment for the BMI before the diabetes diagnosis	1	0.86 (0.78 to 0.94)	0.85 (0.79 to 0.91)	0.87 (0.81 to 0.94)	0.87 (0.80 to 0.96)	0.02
Using beverage intake assessed before the diabetes diagnosis	1	0.82 (0.75 to 0.90)	0.85 (0.79 to 0.92)	0.87 (0.82 to 0.94)	0.90 (0.82 to 0.97)	0.01
Skipping the first food frequency questionnaire after the diagnosis	1	0.94 (0.86 to 1.03)	1.04 (0.98 to 1.11)	0.88 (0.82 to 0.96)	0.99 (0.92 to 1.07)	0.50
Further adjusting for measures of socioeconomic status	1	0.88 (0.80 to 0.98)	0.88 (0.81 to 0.96)	0.90 (0.82 to 0.98)	0.89 (0.80 to 1.00)	0.15
Excluding current and former smokers	1	1.02 (0.87 to 1.21)	0.91 (0.79 to 1.04)	0.91 (0.79 to 1.05)	0.87 (0.74 to 1.03)	0.09
Asymptomatic type 2 diabetes cases only	1	0.87 (0.79 to 0.96)	0.86 (0.79 to 0.93)	0.87 (0.80 to 0.94)	0.88 (0.80 to 0.97)	0.04
Further adjusting for the number of diabetes related symptoms	1	0.86 (0.79 to 0.94)	0.85 (0.79 to 0.92)	0.87 (0.80 to 0.94)	0.87 (0.80 to 0.96)	0.02
Further adjusting for the self-reported levels of glycated hemoglobin HbA_{1c}	1	1.05 (0.91 to 1.21)	0.96 (0.86 to 1.09)	0.85 (0.74 to 0.97)	0.83 (0.70 to 0.98)	0.003

ASB=artificially sweetened beverages; BMI=body mass index; CI=confidence interval; HR=hazard ratio; SSB=sugar-sweetened beverages.

[*] Analyses were adjusted for age (continuous), duration of diabetes mellitus (years), sex (men or women), white ethnicity (yes or no), physical activity (<3.0, 3.0-8.9, 9.0-17.9, 18.0-26.9, ≥27.0 metabolic equivalents-hours/week), smoking status (never, former, current 1-14 cigarettes/day, current ≥15 cigarettes/day), alcohol consumption (0, 0.1-4.9, 5.0-14.9, ≥15.0 g/day), menopausal status and post-menopausal hormone use (pre-menopause, post-menopause (never, former, or current hormone use), or missing; Nurses' Health Study only), family history of type 2 diabetes (yes or no) or myocardial infarction (yes or no), intake of total energy (continuous), the modified Alternative Healthy Eating Index score (fourths), history of hypertension (yes or no) or hypercholesterolemia (yes or no), use of antihypertensive (yes or no) or lipid lowering drug (yes or no), aspirin use (yes or no), diabetes drug use (oral drug only, insulin use, or others), and change in body mass index before to after diabetes diagnosis. Individual beverage consumption was mutually adjusted.

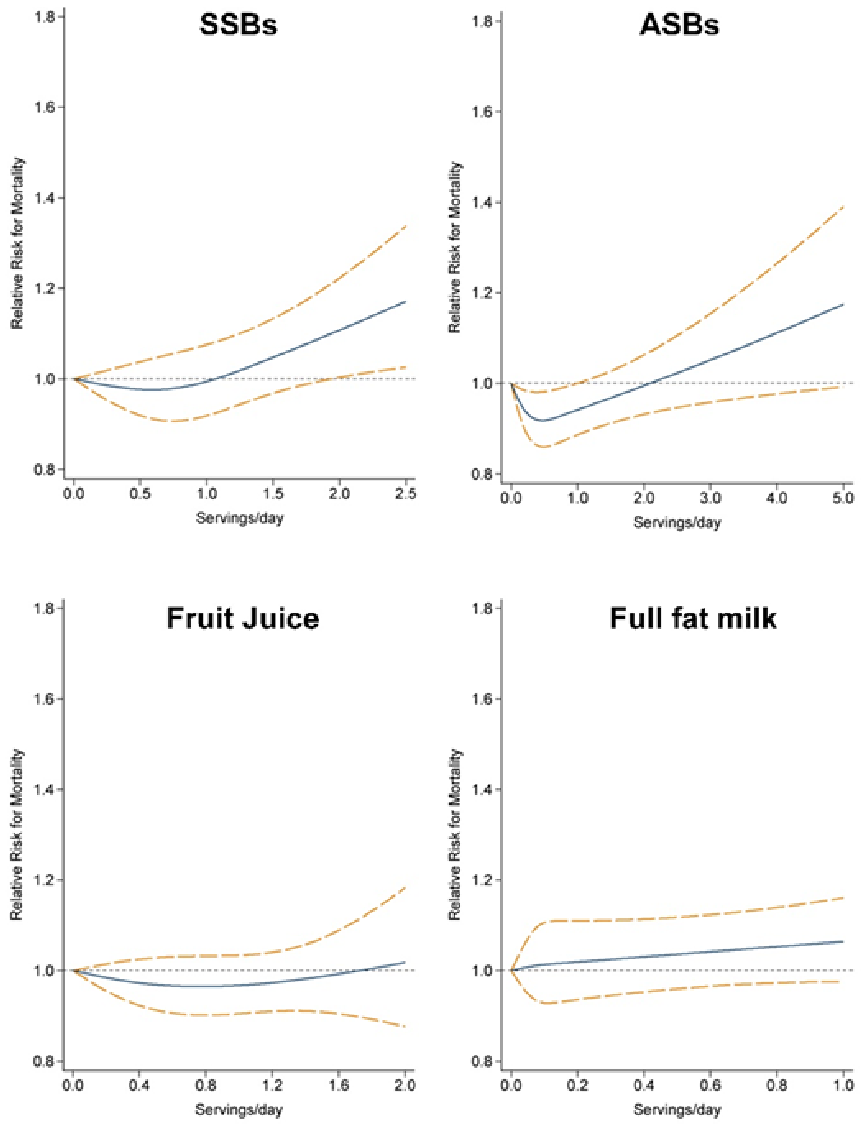

Supplementary Figure 2. Restricted cubic spline analysis of the association between beverage intake assessed the diabetes diagnosis and mortality among adults with type 2 diabetes. Adjusted for age (continuous), duration of diabetes mellitus (years), sex (men or women), white ethnicity (yes or no), physical activity (<3.0, 3.0-8.9, 9.0-17.9, 18.0-26.9, ≥27.0 metabolic equivalents-hours/week), smoking status (never, former, current 1-14 cigarettes/day, current ≥15 cigarettes/day), alcohol consumption (0, 0.1-4.9, 5.0-14.9, ≥15.0 g/day), menopausal status and post-menopausal hormone use (pre-menopause, post-menopause (never, former, or current hormone use), or missing; Nurses' Health Study only), family history of type 2 diabetes (yes or no) or myocardial infarction (yes or no), intake of total energy (continuous), the modified Alternative Healthy Eating Index score (fourths), history of hypertension (yes or no) or hypercholesterolemia (yes or no), use of antihypertensive (yes or no) or lipid lowering drug (yes or no), aspirin use (yes or no), diabetes drug use (oral drug only, insulin use, or others), and change in body mass index before to after diabetes diagnosis. Individual beverage consumption was mutually adjusted.

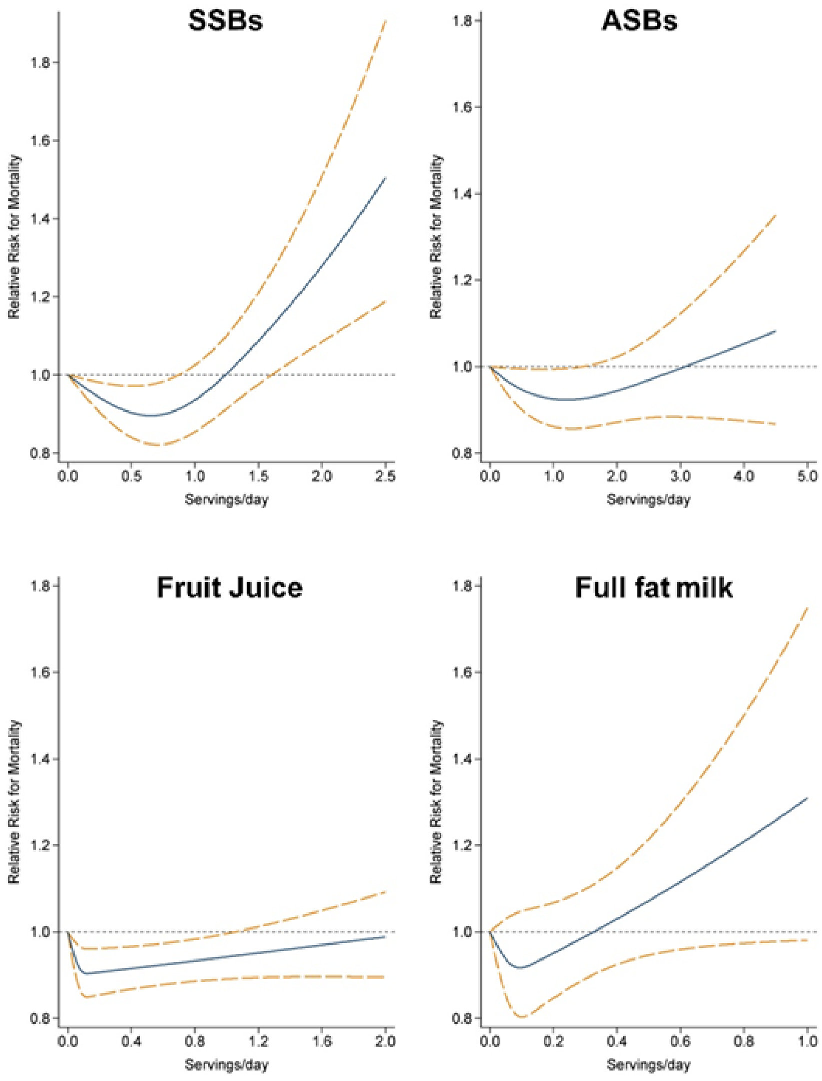

Supplementary Figure 3. Restricted cubic spline analysis of the association between beverage intake (skipping the first food frequency questionnaire after the diagnosis) and mortality among adults with type 2 diabetes. Adjusted for age (continuous), duration of diabetes mellitus (years), sex (men or women), white ethnicity (yes or no), physical activity (<3.0, 3.0-8.9, 9.0-17.9, 18.0-26.9, ≥27.0 metabolic equivalents-hours/week), smoking status (never, former, current 1-14 cigarettes/day, current ≥15 cigarettes/day), alcohol consumption (0, 0.1-4.9, 5.0-14.9, ≥15.0 g/day), menopausal status and post-menopausal hormone use (pre-menopause, post-menopause (never, former, or current hormone use), or missing; Nurses' Health Study only), family history of type 2 diabetes (yes or no) or myocardial infarction (yes or no), intake of total energy (continuous), the modified Alternative Healthy Eating Index score (fourths), history of hypertension (yes or no) or hypercholesterolemia (yes or no), use of antihypertensive (yes or no) or lipid lowering drug (yes or no), aspirin use (yes or no), diabetes drug use (oral drug only, insulin use, or others), and change in body mass index before to after diabetes diagnosis. Individual beverage consumption was mutually adjusted.
